# Medical and obstetric comorbidities and delivery outcomes in overweight and obese parturients: a retrospective analysis

**DOI:** 10.1186/s44158-023-00105-7

**Published:** 2023-06-30

**Authors:** Roi Gat, Eran Hadar, Sharon Orbach-Zinger, Sharon Einav

**Affiliations:** 1 Department of Internal Medicine, Tel Aviv Sourasky Medical Center, Tel Aviv, Israel; 2 Sackler Faculty of Medicine, Tel Aviv University, Tel Aviv, Israel; 3 Rabin Medical Center, Helen Schneider’s Hospital for Women, Petach Tikva, Israel; 4 Department of Anesthesia, Rabin Medical Center, Petach Tikvah, Israel; 5 General Intensive Care Unit, Shaare Zedek Medical Center, Jerusalem, Israel; 6 Faculty of Medicine, Hebrew University, Jerusalem, Israel

**Keywords:** Pregnancy, Overweight, Obesity, BMI, Medical, Obstetric, Comorbidities

## Abstract

**Background:**

Research on obesity in women of reproductive age is heterogeneous in gestational age and body mass index (BMI) classification and focused mostly on pregnancy-related rather than medical comorbidities. We studied the prevalences of pre-pregnancy BMI, chronic maternal and obstetric comorbidities, and delivery outcomes.

**Methods:**

Retrospective analysis of real-time data collected during deliveries in a single tertiary medical center. Pre-pregnancy BMI was classified into seven groups (kg/m^2^): underweight (*BMI* < 18.5), normal weight 1 (18.5 ≤ BMI < 22.5), normal weight 2 (22.5 ≤ BMI < 25.0), overweight 1 (25.0 ≤ BMI < 27.5), overweight 2 (27.5 ≤ BMI < 30.0), obese (30.0 ≤ BMI < 35.0), and morbidly obese (*BMI* ≥ 35.0). Data were collected on maternal demographics, chronic medical and obstetric comorbidities, and delivery outcomes.

**Results:**

Included were 13,726 women aged 18–50 years, with a gestational age of 24^0/7^–41^6/7^ weeks. Pre-pregnancy weights were 61.4% normal, 19.8% overweight, 7.6% obese, and 3.3% morbidly obese. Smoking was more prevalent among morbidly obese than among normal weight women. Obese and morbidly obese women were older and had more diabetes mellitus, hypertension, preeclampsia/eclampsia, and prior cesarean deliveries than normal weight parturients. Obese and morbidly obese women were also less likely to have a non-spontaneous conception, enter labor spontaneously (observed in the full study population and in a subgroup of term parturients), and were more likely to undergo cesarean rather than vaginal delivery. Subgroup analysis of primiparous women yielded similar results.

**Conclusions:**

We identified a potential association between pre-pregnancy obesity and morbid obesity and higher rates of obstetric comorbidities, less natural conception and spontaneous labor, and more cesarean deliveries and adverse delivery outcomes. It remains to be seen if these findings remain after adjustment and whether they are related to obesity, treatment, or both.

**Supplementary Information:**

The online version contains supplementary material available at 10.1186/s44158-023-00105-7.

## Introduction

Obesity, defined by the World Health Organization as body mass index (BMI) ≥ 30 kg/m^2^ [1], is a chronic medical condition with far-reaching health consequences [1, 2]. The prevalence of obesity has been rising almost consistently in recent decades worldwide [3], including among reproductive age women [4].

Obesity has been associated with a myriad of chronic medical conditions [1, 2]. It has also been associated with an increased risk for obstetric comorbidities, cesarean delivery, postoperative complications, poor pregnancy outcomes, and maternal mortality [5] (Supplement 1). However, the research on obese parturients is methodologically heterogenous. Prior studies have determined BMI at different time points during gestation and have used a variety of BMI classifications (Supplement 1). Most have focused solely on pregnancy-related comorbidities and obstetric outcomes [6–9], while chronic medical comorbidities are either unstudied or mentioned only in passing.

The current study was therefore designed to examine the association of pre-pregnancy BMI with medical and obstetric (i.e., current pregnancy) comorbidities and delivery outcomes.

## Methods

We retrospectively analyzed data collected in real time during deliveries in a single medical center and report our findings in accordance with the Strengthening the Reporting of Observational Studies in Epidemiology (STROBE) and Reporting of studies Conducted using Observational Routinely collected Data (RECORD) statements [10, 11]. The study was approved by the institutional review board (0857–19-RMC, submitted December 2019, approved February 2020) with waiver of informed consent.

### Clinical setting

The Rabin Medical Center (RMC) is a 1300-bed tertiary medical center. The RMC labor and delivery ward (L&D) serves approximately 9000 deliveries/year, and the annual cesarean delivery (CD) rate during the study period approximated 23%.

### Participants

We screened the medical files of all women admitted to L&D between 1 July 2012 and 30 June 2016. We sought women aged 18–50 years, admitted for either preterm or term delivery (i.e., gestational age at time of delivery between 24^0/7^ and 41^6/7^ weeks). Women who did not deliver (e.g., L&D admissions for medical observation or treatment) were excluded. We also excluded women admitted for post-term delivery (i.e., gestational age at time of delivery ≥ 42^0/7^ weeks) and for lack of data on pre-pregnancy weight, height, maternal age, or gestational age at time of delivery. Women delivering more than once during the study period were included as distinct cases as their characteristics may have changed in the interim. Follow-up was to the time of hospital discharge.

### Variables

The primary study outcomes were the prevalences of chronic and obstetric maternal morbidity according to pre-pregnancy BMI. Secondary outcomes included delivery characteristics, outcomes, and postpartum complications according to pre-pregnancy BMI among the study population (descriptive), delivery characteristics and outcomes in the subgroup of primiparous women (descriptive), and the distributions and relation between BMIs at the beginning and at the end of pregnancy among study population and in the subgroup of healthy parturients (quantitative).

We collected the following data: maternal age, medical history (e.g., habitual characteristics, any chronic medical condition), height and weight at the beginning of pregnancy and at the time of delivery, obstetric history (e.g., prior pregnancies, deliveries, and outcomes), data regarding current pregnancy (e.g., method of conception, number of fetuses), obstetric complications (e.g., gestational diabetes mellitus, gestational hypertension, preeclampsia/eclampsia), delivery outcomes (e.g., onset of labor [i.e., spontaneous, induction, or CD with no trial of labor], gestational age at birth, mode of delivery), maternal complications during and after delivery (e.g., postpartum hemorrhage), and maternal intensive care unit (ICU) admission.

### Data sources/measurement

Patient admission files, including medical and nursing notes, are fully computerized at the RMC [Chameleon^©^ (electronic health record software, ELAD systems, Tel Aviv, Israel)]. All data were downloaded to a Microsoft Excel^©^ spreadsheet as described elsewhere [12]. Cases were assigned study serial numbers and de-identified.

Data regarding maternal medical and obstetric history, as well as in- and out-patient pregnancy follow-up and complications during the current pregnancy, are drawn automatically from prenatal visits and previous healthcare encounters into the inhospital admission file at the time of admission and are verified with the woman. When prior computerized data are unavailable, the admitting healthcare staff fills the medical file manually, preferably based on written medical documentation. Hence, data regarding height and weight at the beginning of pregnancy were based mainly on prior documentation and rarely on self-report. Weight at the end of pregnancy was documented in real time by the healthcare staff as were obstetric complications and delivery outcomes.

### Bias and confounding

We included all consecutive women within a predefined time frame in order to minimize selection bias and studied more than 1 year in order to ensure our data does not reflect a limited time period. A subgroup of primiparas was studied in order to verify that parity did not influence the relation between BMI and delivery outcomes. Gestational age was adjusted for when studying the relationship between BMI and onset of delivery.

### Study size

As the main study outcome was observational, the sample size was planned to provide stable estimates for the prevalence of medical and obstetric comorbidities according to pre-pregnancy BMI based on prior studies [8, 9].

### Quantitative variables

BMI was calculated as weight/height^2^ (kg/m^2^) and divided into seven groups: underweight (*BMI* < 18.50), normal weight 1 (18.50 ≤ BMI < 22.50), normal weight 2 (22.50 ≤ BMI < 25.00), overweight 1 (25.00 ≤ BMI < 27.50), overweight 2 (27.50 ≤ BMI < 30.00), obesity (30.00 ≤ BMI < 35.00), and morbid obesity (35.00 ≤ BMI) [13]. Parity was divided into three groups: primiparous (1st delivery), multiparous (2nd–4th delivery), and grand-multiparous (5th delivery and beyond) women [14]. Gestational age at birth was divided into term (37^0/7^–41^6/7^ weeks), late preterm (34^0/7^–36^6/7^ weeks), and preterm (24^0/7^–33^6/7^ weeks) [15].

Type 1 diabetes mellitus (DM) and type 2 DM were unified into a single variable termed “pregestational DM.” Preeclampsia, superimposed preeclampsia, HELLP (hemolysis, elevated liver enzymes, and low platelets) syndrome, and eclampsia were unified into a single variable termed “preeclampsia/eclampsia.”

### Statistical analysis

Pre-pregnancy and end of pregnancy BMIs were calculated for each case. Cases with data on pre-pregnancy BMI but missing data regarding other variables (e.g., method of conception, mode of delivery) were included. Illogical values (e.g., age = 0 or greater than 60 years, height ≤ 1.3 m) were imputed as missing data and excluded from the analysis. Included cases (i.e., the study population) and excluded cases were compared in order to examine selection bias.

After cleaning, the data were analyzed using SAS 9.4 (SAS Institute Inc., Cary, NC, USA). Descriptive statistics included counts, percentages, averages with their standard deviations (SDs), medians with their interquartile ranges, and ranges. Percentages were all calculated from the cohort (or relevant sub-cohort) as a whole rather than from existing data. The precision of the estimates for each variable is presented as the 95% confidence interval (CI), and group comparisons are based on the precision estimates. Odds ratios (ORs) and Wald CIs were calculated for induction of labor and CD with no trial of labor by pre-pregnancy BMI and post hoc for end of pregnancy BMIs. The onset of labor was also stratified post hoc by gestational age in order to study its relationship with BMI at the beginning and end of pregnancy. Finally, subgroup analysis of primiparous women was conducted to further clarify the relation between the mode of delivery (MOD) and BMI.

## Results

During the study period 35,905 women delivered at the RMC. Overall, 13,726 women fulfilled eligibility criteria and were included in the analysis (Supplement 2). Among the excluded cases (*n* = 22,179), the proportion of women with medical and obstetric comorbidities and the proportion of women undergoing CD were lower than among the included cases (Supplement 3). The proportion of missing data in the included cohort was lower than 0.5% in all but eight variables (Supplement 4).

The average age of the included parturients was 31.4 ± 5.2 years, and the average gestational age at delivery was 39^0/7^ weeks ± 13 days. Further details on the demographics, obstetric characteristics, and comorbidities of the cohort as a whole are presented in Supplements 3 and 5.

### Pre-pregnancy BMI (Supplement 6)

Overall, 7.8% of the study population were classified as underweight (*n* = 1074), 39.9% as normal weight 1 (*n* = 5473), 21.5% as normal weight 2 (*n* = 2957), 12.6% as overweight 1 (*n* = 1734), 7.2% as overweight 2 (*n* = 990), 7.6% as obese (*n* = 1039), and 3.3% as morbidly obese (*n* = 459).

### Primary outcome

#### Demographic characteristics and chronic comorbidities by pre-pregnancy BMI (Table 1, Supplement 6)

**Table 1:** Substance abuse and medical background characteristics by pre-pregnancy BMI

	**Pre-pregnancy BMI**													
	**Underweight (** ***N*** ** = 1074)**		**Normal weight 1 (** ***N*** ** = 5473)**		**Normal weight 2 (** ***N*** ** = 2957)**		**Overweight 1 (** ***N*** ** = 1734)**		**Overweight 2 (** ***N*** ** = 990)**		**Obesity (** ***N*** ** = 1039)**		**Morbid obesity (** ***N*** ** = 459)**	
	***N*** ** (%)**	***CI*** ** [%]**	***N*** ** (%)**	***CI*** ** [%]**	***N*** ** (%)**	***CI*** ** [%]**	***N*** ** (%)**	***CI*** ** [%]**	***N*** ** (%)**	***CI*** ** [%]**	***N*** ** (%)**	***CI*** ** [%]**	***N*** ** (%)**	***CI*** ** [%]**
**Smoking**	82 (7.64%)	6.12–9.39∆	296 (5.41%)	4.82–6.04	157 (5.31%)	4.53–6.18	86 (4.96%)	3.99–6.09	55 (5.56%)	4.21–7.17	67 (6.45%)	5.03–8.12	41 (8.93%)	6.49–11.92▲
**Alcohol consumption**	5 (0.47%)	0.15–1.08	16 (0.29%)	0.17–0.47	8 (0.27%)	0.12–0.53	5 (0.29%)	0.09–0.67	3 (0.30%)	0.06–0.88	3 (0.29%)	0.06–0.84	2 (0.44%)	0.05–1.57
**Drug abuse**	7 (0.65%)	0.26–1.34	13 (0.24%)	0.13–0.41	15 (0.51%)	0.28–0.84	5 (0.29%)	0.09–0.67	4 (0.40%)	0.11–1.03	3 (0.29%)	0.06–0.84	1 (0.22%)	0.01–1.21
**Pregestational DM (type 1 or 2)**	32 (2.98%)	2.05–4.18■	274 (5.01%)	4.44–5.62	182 (6.15%)	5.32–7.08	129 (7.44%)	6.25–8.78∆	60 (6.06%)	4.66–7.73	109 (10.49%)	8.69–12.52▲	65 (14.16%)	11.10–17.69▲
**Pregestational HTN**	27 (2.51%)	1.66–3.64	188 (3.44%)	2.97–3.95	120 (4.06%)	3.38–4.83	99 (5.71%)	4.66–6.91∆	59 (5.96%)	4.57–7.62∆	79 (7.60%)	6.07–9.39▲	44 (9.59%)	7.05–12.65▲
**Cardiac disease (any)** ^a^	16 (1.49%)	0.85–2.41	105 (1.92%)	1.57–2.32	63 (2.13%)	1.64–2.72	63 (3.63%)	2.80–4.62▲	24 (2.42%)	1.56–3.59	27 (2.60%)	1.72–3.76	15 (3.27%)	1.84–5.33
**Pulmonary disease (any)** ^b^	13 (1.21%)	0.65–2.06	63 (1.15%)	0.89–1.47	36 (1.22%)	0.85–1.68	19 (1.10%)	0.66–1.71	15 (1.52%)	0.85–2.49	21 (2.02%)	1.26–3.07	11 (2.40%)	1.20–4.25
**Asthma**	9 (0.84%)	0.38–1.58	60 (1.10%)	0.84–1.41	35 (1.18%)	0.83–1.64	17 (0.98%)	0.57–1.57	15 (1.52%)	0.85–2.49	21 (2.02%)	1.26–3.07	11 (2.40%)	1.20–4.25
**Renal disease**	0 (0.00%)	0.00–0.34	9 (0.16%)	0.08–0.31	3 (0.10%)	0.02–0.30	4 (0.23%)	0.06–0.59	1 (0.10%)	0.00–0.56	1 (0.10%)	0.00–0.54	0 (0.00%)	0.00–0.80
**Past kidney transplant**	0 (0.00%)	0.00–0.34	2 (0.04%)	0.00–0.13	2 (0.07%)	0.01–0.24	4 (0.23%)	0.06–0.59	2 (0.20%)	0.02–0.73	0 (0.00%)	0.00–0.35	1 (0.22%)	0.01–1.21
**Gastrointestinal disease (any)** ^c^	7 (0.65%)	0.26–1.34	23 (0.42%)	0.27–0.63	18 (0.61%)	0.36–0.96	2 (0.12%)	0.01–0.42	3 (0.30%)	0.06–0.88	4 (0.38%)	0.10–0.98	1 (0.22%)	0.01–1.21
**Hepatobiliary disease (any)** ^d^	0 (0.00%)	0.00–0.34	4 (0.07%)	0.02–0.26	1 (0.03%)	0.00–0.31	2 (0.12%)	0.01–0.63	1 (0.10%)	0.00–0.93	3 (0.29%)	0.02–1.23	0 (0.00%)	0.00–0.80
**Rheumatic disease (any)** ^e^	23 (2.14%)	1.36–3.20	133 (2.43%)	2.04–2.87	91 (3.08%)	2.48–3.77	52 (3.00%)	2.25–3.91	38 (3.84%)	2.73–5.23	45 (4.33%)	3.18–5.75∆	16 (3.49%)	2.01–5.60
**Hypothyroidism**	15 (1.40%)	0.78–2.29	101 (1.85%)	1.51–2.24	76 (2.57%)	2.03–3.21	38 (2.19%)	1.56–3.00	27 (2.73%)	1.80–3.94	40 (3.85%)	2.76–5.21∆	15 (3.27%)	1.84–5.33
**Hematologic disease (any)** ^f^	6 (0.56%)	0.21–1.21	36 (0.66%)	0.46–0.91	18 (0.61%)	0.36–0.96	10 (0.58%)	0.28–1.06	6 (0.61%)	0.22–1.31	5 (0.48%)	0.16–1.12	3 (0.65%)	0.13–1.90
**Hypercoagulopathy (any)** ^g^	8 (0.74%)	0.32–1.46	84 (1.53%)	1.23–1.90	37 (1.25%)	0.88–1.72	32 (1.85%)	1.27–2.60	14 (1.41%)	0.78–2.36	19 (1.83%)	1.10–2.84	10 (2.18%)	1.05–3.97
**Malignancy — past or present (any)** ^h^	18 (1.68%)	1.00–2.64	93 (1.70%)	1.37–2.08	52 (1.76%)	1.32–2.30	34 (1.96%)	1.36–2.73	21 (2.12%)	1.32–3.22	18 (1.73%)	1.03–2.72	7 (1.53%)	0.62–3.12
**CNS disorders (any)** ^i^	6 (0.56%)	0.21–1.21	49 (0.90%)	0.66–1.18	26 (0.88%)	0.58–1.29	21 (1.21%)	0.75–1.85	9 (0.91%)	0.42–1.72	12 (1.15%)	0.60–2.01	3 (0.65%)	0.13–1.90

*BMI* Body mass index, *N* Number, *CI* 95% confidence interval, *DM* Diabetes mellitus, *HTN* Hypertension, *CNS* Central nervous system

*∆*Higher than normal weight 1

▲higher than normal weight 1 and normal weight 2

□lower than normal weight 1

■lower than normal weight 1 and normal weight 2

^a^either cardiomyopathy, coarctation of aorta, congenital heart defect, congestive heart failure, ischemic heart disease, rheumatic heart disease, or valvulopathy

^b^either asthma, chronic obstructive lung disease, primary pulmonary hypertension, restrictive lung disease, or tuberculosis

^c^either celiac disease/celiac sprue, inflammatory bowel disease, or irritable bowel syndrome

^d^either choledocholithiasis, chronic pancreatitis, or chronic liver disease

^e^either familial Mediterranean fever, hypothyroidism, hyperthyroidism, or systemic lupus erythematosus

^f^chronic disorder of either platelets, red blood cells, or white blood cells

^g^either acquired or congenital

^h^either past or present malignancy (not including basal cell and squamous cell carcinomas of skin)

^i^either Alzheimer’s disease, ataxia, benign intracranial hypertension, cerebral palsy, congenital malformations of central nervous system, epilepsy, multiple sclerosis, past or present cerebrovascular accident, past or present transient ischemic attack, Parkinson’s disease or poliomyelitis

**Fig. 1:**
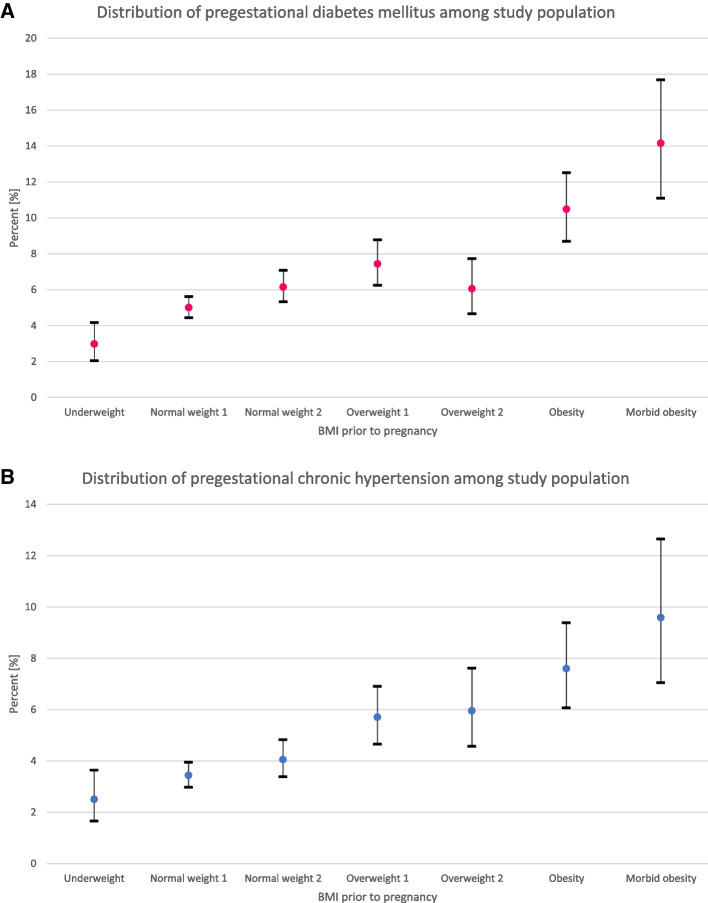
**A** Distribution of pregestational diabetes mellitus among study population. **B** Distribution of pregestational hypertension among study population

Maternal age and height were similar among study groups. There were more smokers among pre-pregnancy morbidly obese women than among women with a normal pre-pregnancy BMI. Pregestational DM and pregestational HTN were significantly more prevalent among obese and morbidly obese women, and their prevalences increased with increasing BMI when compared to women with normal pre-pregnancy BMI (Fig. 1).

#### Obstetric history and current pregnancy characteristics by pre-pregnancy BMI (Table 2)

**Table 2:** Parity, prior cesarean delivery, and characteristics of the current pregnancy by pre-pregnancy BMI

	**Pre-pregnancy BMI**													
	**Underweight (** ***N*** ** = 1074)**		**Normal weight 1 (** ***N*** ** = 5473)**		**Normal weight 2 (** ***N*** ** = 2957)**		**Overweight 1 (** ***N*** ** = 1734)**		**Overweight 2 (** ***N*** ** = 990)**		**Obesity (** ***N*** ** = 1039)**		**Morbid obesity (** ***N*** ** = 459)**	
	***N*** ** (%)**	***CI*** ** [%]**	***N*** ** (%)**	***CI*** ** [%]**	***N*** ** (%)**	***CI*** ** [%]**	***N*** ** (%)**	***CI*** ** [%]**	***N*** ** (%)**	***CI*** ** [%]**	***N*** ** (%)**	***CI*** ** [%]**	***N*** ** (%)**	***CI*** ** [%]**
**Parity**														
**Primiparous**	525 (48.88%)	45.85–51.92▲	2335 (42.66%)	41.35–43.99	1053 (35.61%)	33.88–37.37□	587 (33.85%)	31.63–36.13□	309 (31.21%)	28.33–34.20□	335 (32.24%)	29.41–35.18□	157 (34.20%)	29.87–38.74□
**Multiparous**	516 (48.04%)	45.02–51.08■	2971 (54.28%)	52.95–55.61	1767 (59.76%)	57.96–61.53∆	1044 (60.21%)	57.86–62.52∆	602 (60.81%)	57.69–63.86∆	600 (57.75%)	54.68–60.77	267 (58.17%)	53.51–62.73
**Grand multiparous**	33 (3.07%)	2.12–4.29	166 (3.03%)	2.59–3.52	137 (4.63%)	3.90–5.45∆	102 (5.88%)	4.82–7.10∆	79 (7.98%)	6.37–9.85▲	103 (9.91%)	8.16–11.89▲	35 (7.63%)	5.37–10.45∆
**Past cesarean delivery**														
**None**	975 (90.78%)	88.89–92.45	4840 (88.43%)	87.56–89.27	2518 (85.15%)	83.82–86.42□	1442 (83.16%)	81.31–84.89□	802 (81.01%)	78.43–83.41■	829 (79.79%)	77.22–82.19■	356 (77.56%)	73.46–81.30■
**≥ 1**	99 (9.22%)	7.55–11.11	633 (11.57%)	10.73–12.44	439 (14.85%)	13.58–16.18∆	292 (16.84%)	15.11–18.69∆	188 (18.99%)	16.59–21.57▲	210 (20.21%)	17.81–22.78▲	103 (22.44%)	18.70–26.54▲
**Method of conception**														
**Spontaneous**	908 (84.54%)	82.24–86.66	4628 (84.56%)	83.58–85.51	2488 (84.14%)	82.77–85.44	1437 (82.87%)	81.01–84.62	814 (82.22%)	79.70–84.56	846 (81.42%)	78.92–83.75	354 (77.12%)	73.00–80.89■
**Non spontaneous**	85 (7.91%)	6.37–9.69	410 (7.49%)	6.81–8.22	238 (8.05%)	7.09–9.09	145 (8.36%)	7.10–9.77	95 (9.60%)	7.83–11.60	120 (11.55%)	9.67–13.65▲	69 (15.03%)	11.89–18.64▲
**Number of fetuses**														
**Singleton**	1037 (96.55%)	95.28–97.56	5308 (96.99%)	96.50–97.42	2860 (96.72%)	96.01–97.33	1669 (96.25%)	95.25–97.10	952 (96.16%)	94.77–97.27	1022 (98.36%)	97.39–99.04	435 (94.77%)	92.32–96.62
**Multiple**	36 (3.35%)	2.36–4.61	162 (2.96%)	2.53–3.44	96 (3.25%)	2.64–3.95	62 (3.58%)	2.75–4.56	38 (3.84%)	2.73–5.23	17 (1.64%)	0.96–2.61	24 (5.23%)	3.38–7.68

*BMI* Body mass index, *N* Number, *CI* 95% confidence interval

∆higher than normal weight 1

▲higher than normal weight 1 and normal weight 2

□lower than normal weight 1

■lower than normal weight 1 and normal weight 2

The proportion of grand-multiparous women was higher among pre-pregnancy overweight 2 and obese women than among women with a normal pre-pregnancy BMI. The proportion of women with a prior CD also increased with increasing BMI. Obese and morbidly obese women were less likely to conceive spontaneously than women with a normal pre-pregnancy BMI.

#### Obstetric morbidities by pre-pregnancy BMI (Table 3)

**Table 3:** Obstetric complications by pre-pregnancy BMI

	**Pre-pregnancy BMI**													
	**Underweight (** ***N*** ** = 1074)**		**Normal weight 1 (** ***N*** ** = 5473)**		**Normal weight 2 (** ***N*** ** = 2957)**		**Overweight 1 (** ***N*** ** = 1734)**		**Overweight 2 (** ***N*** ** = 990)**		**Obesity (** ***N*** ** = 1039)**		**Morbid obesity (** ***N*** ** = 459)**	
	***N*** ** (%)**	***CI*** ** [%]**	***N*** ** (%)**	***CI*** ** [%]**	***N*** ** (%)**	***CI*** ** [%]**	***N*** ** (%)**	***CI*** ** [%]**	***N*** ** (%)**	***CI*** ** [%]**	***N*** ** (%)**	***CI*** ** [%]**	***N*** ** (%)**	***CI*** ** [%]**
**Gestational DM**	45 (4.19%)	3.07–5.57	316 (5.77%)	5.17–6.42	313 (10.59%)	9.50–11.75∆	277 (15.97%)	14.28–17.79▲	201 (20.30%)	17.84–22.95▲	253 (24.35%)	21.77–27.08▲	143 (31.15%)	26.94–35.61▲
**Gestational HTN**	30 (2.79%)	1.81–3.78	164 (3.00%)	2.54–3.45	120 (4.06%)	3.35–4.70	86 (4.96%)	3.94–5.98∆	64 (6.46%)	4.93–8.00▲	92 (8.85%)	7.12–10.58▲	56 (12.20%)	9.20–15.21▲
**Preeclampsia/eclampsia**	30 (2.79%)	1.89–3.96	109 (1.99%)	1.64–2.40	88 (2.98%)	2.39–3.65	57 (3.29%)	2.50–4.24∆	43 (4.34%)	3.16–5.81∆	62 (5.97%)	4.61–7.58▲	46 (10.02%)	7.43–13.14▲
**Intrauterine growth restriction**	68 (6.33%)	4.95–7.96▲	175 (3.20%)	2.75–3.70	70 (2.37%)	1.85–2.98	45 (2.60%)	1.90–3.46	30 (3.03%)	2.05–4.30	17 (1.64%)	0.96–2.61□	10 (2.18%)	1.05–3.97
**Chorioamnionitis**	2 (0.19%)	0.02–0.67	4 (0.07%)	0.02–0.19	8 (0.27%)	0.12–0.53	4 (0.23%)	0.06–0.59	3 (0.30%)	0.06–0.88	2 (0.19%)	0.02–0.69	2 (0.44%)	0.05–1.57
**Intrahepatic cholestasis of pregnancy**	8 (0.74%)	0.32–1.46	32 (0.58%)	0.40–0.82	16 (0.54%)	0.31–0.88	10 (0.58%)	0.28–1.06	7 (0.71%)	0.28–1.45	7 (0.67%)	0.27–1.38	7 (1.53%)	0.62–3.12
**Placental abruption**	7 (0.65%)	0.26–1.34	25 (0.46%)	0.30–0.67	9 (0.30%)	0.14–0.58	8 (0.46%)	0.20–0.91	7 (0.71%)	0.28–1.45	7 (0.67%)	0.27–1.38	1 (0.22%)	0.01–1.21
**Deep vein thrombosis**	4 (0.37%)	0.10–0.95	9 (0.16%)	0.08–0.31	7 (0.24%)	0.10–0.49	4 (0.23%)	0.06–0.59	0 (0.00%)	0.00–0.37	3 (0.29%)	0.06–0.84	4 (0.87%)	0.24–2.22
**Pulmonary embolism**	0 (0.00%)	0.00–0.34	5 (0.09%)	0.03–0.21	4 (0.14%)	0.04–0.35	1 (0.06%)	0.00–0.32	1 (0.10%)	0.00–0.56	3 (0.29%)	0.06–0.84	1 (0.22%)	0.01–1.21

*BMI* Body mass index, *N* Number, *CI* 95% confidence interval, *DM* Diabetes mellitus, *HTN* hypertension; preeclampsia/eclampsia, a composite variable of preeclampsia, superimposed preeclampsia, *HELLP* (hemolysis, elevated liver enzymes, and low platelets) syndrome and eclampsia

∆higher than normal weight 1

▲higher than normal weight 1 and normal weight 2

□lower than normal weight 1

■lower than normal weight 1 and normal weight 2

Gestational DM was significantly more prevalent among overweight, obese, and morbidly obese women than among women with a normal pre-pregnancy BMI. Gestational hypertension was significantly more prevalent among pre-pregnancy overweight 2, obese, and morbidly obese women than among women with a normal pre-pregnancy BMI. The prevalence of preeclampsia/eclampsia was also higher in obese and morbidly obese women when compared to women with a normal pre-pregnancy BMI. As BMI increased beyond normal weight 1, the preval1ence of gestational DM, gestational hypertension, and preeclampsia/eclampsia increased constantly.

### Secondary outcomes

#### Delivery characteristics and postpartum complications by pre-pregnancy BMI (Table 4)

**Table 4:** Gestational age at delivery, onset and mode of delivery, urgency of cesarean delivery, and peripartum maternal complications by pre-pregnancy BMI

	**Pre-pregnancy BMI**													
	**Underweight (** ***N*** ** = 1074)**		**Normal weight 1 (** ***N*** ** = 5473)**		**Normal weight 2 (** ***N*** ** = 2957)**		**Overweight 1 (** ***N*** ** = 1734)**		**Overweight 2 (** ***N*** ** = 990)**		**Obesity (** ***N*** ** = 1039)**		**Morbid obesity (** ***N*** ** = 459)**	
	***N*** ** (%)**	***CI*** ** [%]**	***N*** ** (%)**	***CI*** ** [%]**	***N*** ** (%)**	***CI*** ** [%]**	***N*** ** (%)**	***CI*** ** [%]**	***N*** ** (%)**	***CI*** ** [%]**	***N*** ** (%)**	***CI*** ** [%]**	***N*** ** (%)**	***CI*** ** [%]**
**Gestational age at delivery (wk)**	1074 (100%)	39^0/7^ ± 13d^a^	5473 (100%)	39^1/7^ ± 13d^a^	2957 (100%)	39^1/7^ ± 13d^a^	1734 (100%)	39^1/7^ ± 12d^a^	990 (100%)	38^6/7^ ± 13d^a^	1039 (100%)	38^6/7^ ± 12d^a^	459 (100%)	38^4/7^ ± 15d^a^
**Gestational age at delivery (wk)**														
**Preterm (24**^**0/7**^**–33**^**6/7**^**)**	28 (2.61%)	1.74–3.75	78 (1.43%)	1.13–1.78	58 (1.96%)	1.49–2.53	18 (1.04%)	0.62–1.64	16 (1.62%)	0.93–2.61	11 (1.06%)	0.53–1.89	15 (3.27%)	1.84–5.33∆
**Late preterm (34**^**0/7**^**–36**^**6/7**^**)**	72 (6.70%)	5.28–8.37	319 (5.83%)	5.22–6.48	176 (5.95%)	5.13–6.87	118 (6.81%)	5.66–8.09	75 (7.58%)	6.01–9.40	79 (7.60%)	6.07–9.39	31 (6.75%)	4.63–9.45
**Term (37**^**0/7**^**–41**^**6/7**^**)**	974 (90.69%)	88.79–92.36	5076 (92.75%)	92.03–93.42	2723 (92.09%)	91.05–93.03	1598 (92.16%)	90.79–93.38	899 (90.81%)	88.83–92.53	949 (91.34%)	89.46–92.98	413 (89.98%)	86.86–92.57
**Onset of labor**														
**Spontaneous**	634 (59.03%)	56.02–61.99	3153 (57.61%)	56.29–58.92	1555 (52.59%)	50.77–54.40□	840 (48.44%)	46.07–50.82□	413 (41.72%)	38.62–44.86■	382 (36.77%)	33.83–39.78■	133 (28.98%)	24.86–33.36■
**Induction**	272 (25.33%)	22.75–28.04	1380 (25.21%)	24.07–26.39	845 (28.58%)	26.95–30.24∆	485 (27.97%)	25.87–30.15	328 (33.13%)	30.20–36.16∆	357 (34.36%)	31.47–37.34▲	160 (34.86%)	30.50–39.41▲
**CD — no trial of labor**	117 (10.89%)	9.09–12.91	657 (12.00%)	11.15–12.89	408 (13.80%)	12.57–15.09	285 (16.44%)	14.72–18.27∆	192 (19.39%)	16.97–22.00▲	224 (21.56%)	19.09–24.19▲	132 (28.76%)	24.66–33.14▲
**Mode of delivery**														
**Vaginal delivery**	785 (73.09%)	70.33–75.72	3,887 (71.02%)	69.80–72.22	2,051 (69.36%)	67.66–71.02	1129 (65.11%)	62.81–67.35■	627 (63.33%)	60.24–66.34■	616 (59.29%)	56.23–62.29■	231 (50.33%)	45.65–54.99■
**Operative vaginal delivery**	96 (8.94%)	7.30–10.81	533 (9.74%)	8.97–10.55	253 (8.56%)	7.57–9.62	142 (8.19%)	6.94–9.58	69 (6.97%)	5.46–8.74□	76 (7.31%)	5.81–9.07	20 (4.36%)	2.68–6.65■
**Cesarean delivery**	193 (17.97%)	15.72–20.40	1050 (19.19%)	18.15–20.25	653 (22.08%)	20.60–23.62^∆^	463 (26.70%)	24.63–28.85▲	294 (29.70%)	26.86–32.65▲	347 (33.40%)	30.53–36.36▲	207 (45.10%)	40.48–49.78▲
**Urgency of cesarean delivery**														
**Elective**	79 (40.93%)	33.92–48.22	504 (48.00%)	44.94–51.07	322 (49.31%)	45.41–53.22	249 (53.78%)	49.12–58.39	163 (55.44%)	49.56–61.21	182 (52.45%)	47.05–57.81	99 (47.83%)	40.85–54.86
**Semi-elective**	19 (9.84%)	6.03–14.95	55 (5.24%)	3.97–6.76	52 (7.96%)	6.00–10.31	28 (6.05%)	4.06–8.62	21 (7.14%)	4.48–10.71	24 (6.92%)	4.48–10.12	19 (9.18%)	5.62–13.96
**Urgent**	94 (48.70%)	41.46–55.99	486 (46.29%)	43.24–49.36	277 (42.42%)	38.59–46.31	184 (39.74%)	35.25–44.36	106 (36.05%)	30.56–41.83□	139 (40.06%)	34.86–45.42	87 (42.03%)	35.22–49.07
**Postpartum hemorrhage**	37 (3.45%)	2.44–4.72	218 (3.98%)	3.48–4.54	130 (4.40%)	3.69–5.20	64 (3.69%)	2.85–4.69	40 (4.04%)	2.90–5.46	26 (2.50%)	1.64–3.65	12 (2.61%)	1.36–4.52
**ICU admission**	7 (0.65%)	0.26–1.34	21 (0.38%)	0.24–0.59	19 (0.64%)	0.39–1.00	15 (0.87%)	0.48–1.42	4 (0.40%)	0.11–1.03	6 (0.58%)	0.21–1.25	4 (0.87%)	0.24–2.22

*BMI* Body mass index, *N* Number, *CI* 95% confidence interval, *wk* week, *d* day, *CD* Cesarean delivery, *ICU* Intensive care unit

^a^Mean ± standard deviation

∆higher than normal weight 1

▲higher than normal weight 1 and normal weight 2

□lower than normal weight 1

■lower than normal weight 1 and normal weight 2

No differences were observed in gestational age at time of delivery in the different BMI groups. However, with increasing BMI, the rate of spontaneous onset of delivery decreased and the rate of both induction of labor and CD with no trial of labor increased significantly. This finding was attributed to women at term delivery with a pre-pregnancy BMI category of overweight 2 or more (Supplements 7–9). Onset of labor did not differ across the various pre-pregnancy BMI groups among women with preterm deliveries (Supplement 7). Similar findings were observed regarding onset of labor when stratified to end of pregnancy BMI groups (Supplements 10–12).

The rates of vaginal delivery decreased, and the rates of CD increased constantly with increasing BMI in overweight, obese, and morbidly obese women when compared to normal weight parturients. Ultimately, morbidly obese women were more than twice as likely to undergo CD, and in particular CD with no trial of labor, when compared to women who started their pregnancy with a normal BMI. The rates of postpartum hemorrhage and ICU admission were constant regardless of BMI.

#### Subgroup analysis of primiparous women (Table 5)

**Table 5:** Mode of delivery, cesarean delivery characteristics, and neonatal birth weight in primiparous parturients by pre-pregnancy BMI

	**Pre-pregnancy BMI**													
	**Underweight (** ***N*** ** = 525)**		**Normal weight 1 (** ***N*** ** = 2335)**		**Normal weight 2 (** ***N*** ** = 1053)**		**Overweight 1 (** ***N*** ** = 587)**		**Overweight 2 (** ***N*** ** = 309)**		**Obesity (** ***N*** ** = 335)**		**Morbid obesity (** ***N*** ** = 157)**	
	***N*** ** (%)**	***CI*** ** [%]**	***N*** ** (%)**	***CI*** ** [%]**	***N*** ** (%)**	***CI*** ** [%]**	***N*** ** (%)**	***CI*** ** [%]**	***N*** ** (%)**	***CI*** ** [%]**	***N*** ** (%)**	***CI*** ** [%]**	***N*** ** (%)**	***CI*** ** [%]**
**Mode of delivery**														
**Vaginal delivery**	358 (68.19%)	64.02–72.16	1497 (64.11%)	62.13–66.06	621 (58.97%)	55.93–61.96□	317 (54.00%)	49.87–58.09□	165 (53.40%)	47.66–59.07□	173 (51.64%)	46.15–57.11□	60 (38.22%)	30.59–46.30■
**Operative vaginal delivery**	78 (14.86%)	11.92–18.19	404 (17.30%)	15.79–18.90	188 (17.85%)	15.59–20.30	98 (16.70%)	13.77–19.96	56 (18.12%)	13.99–22.88	54 (16.12%)	12.35–20.50	17 (10.83%)	6.44–16.77
**CD**	89 (16.95%)	13.84–20.44	433 (18.54%)	16.99–20.18	244 (23.17%)	20.65–25.84∆	172 (29.30%)	25.65–33.17∆	88 (28.48%)	23.51–33.86∆	108 (32.24%)	27.26–37.53▲	80 (50.96%)	42.86–59.01▲
**Urgency of CD**														
**Elective**	22 (24.72%)	16.19–35.00	144 (33.26%)	28.83–37.91	74 (30.33%)	24.63–36.52	67 (38.95%)	31.62–46.67	33 (37.50%)	27.40–48.47	40 (37.04%)	27.94–46.86	21 (26.25%)	17.04–37.29
**Semi-elective**	13 (14.61%)	8.01–23.68	24 (5.54%)	3.58–8.14	21 (8.61%)	5.41–12.86	5 (2.91%)	0.95–6.65	8 (9.09%)	4.01–17.13	4 (3.70%)	1.02–9.21	7 (8.75%)	3.59–17.20
**Urgent**	53 (59.55%)	48.62–69.83	261 (60.28%)	55.50–64.92	148 (60.66%)	54.22–66.83	99 (57.56%)	49.80–65.05	46 (52.27%)	41.35–63.04	64 (59.26%)	49.38–68.62	52 (65.00%)	53.52–75.33
**Indication for CD**														
**Malpresentation**	28 (31.46%)	22.03–42.17	112 (25.87%)	21.80–30.26	36 (14.75%)	10.55–19.84□	35 (20.35%)	14.60–27.15	13 (14.77%)	8.11–23.94	13 (12.04%)	6.57–19.70□	7 (8.75%)	3.59–17.20□
**Failed induction**	7 (7.87%)	3.22–15.54	24 (5.54%)	3.58–8.14	24 (9.84%)	6.40–14.28	14 (8.14%)	4.52–13.28	9 (10.23%)	4.78–18.53	11 (10.19%)	5.20–17.49	13 (16.25%)	8.95–26.18∆
**Suspected macrosomia**	1 (1.12%)	0.03–6.10	13 (3.00%)	1.61–5.08	11 (4.51%)	2.27–7.92	12 (6.98%)	3.66–11.87	8 (9.09%)	4.01–17.13	6 (5.56%)	2.07–11.70	10 (12.50%)	6.16–21.79∆
**Actual birth weight in women whose indication for CD was suspected macrosomia (mean ± SD) (g)**	4604 ± 0.0		4078 ± 244.8		4048 ± 284.2		4012 ± 325.4		4086 ± 365.4		4243 ± 607.1		3892 ± 293.7	

*BMI* Body mass index, *N* Number, *CI* 95% confidence interval, *CD* cesarean delivery, *SD* Standard deviation, *g* gram

∆higher than normal weight 1

▲higher than normal weight 1 and normal weight 2

□lower than normal weight 1

■lower than normal weight 1 and normal weight 2

When compared to primiparous women with a normal pre-pregnancy weight 1, overweight, obese, and morbidly obese primiparas were increasingly less likely to undergo a vaginal delivery and increasingly more likely to undergo CD.

The indication for CD was less commonly malpresentation and more commonly failed induction of labor or suspected macrosomia among morbidly obese primiparas when compared to pre-pregnancy normal weight 1 primiparas (Supplement 13). Yet, the actual neonatal birth weight of morbidly obese women whose indication for CD was suspected macrosomia was below 4.0 kg and lower than that of normal and underweight women.

#### Distribution of BMIs

The distribution of BMIs at the beginning and at the end of pregnancy among the study population as a whole and in the subgroup of healthy parturients is presented in Supplement 14. No differences were observed.

#### Unadjusted relation between BMI at the beginning and at the end of pregnancy

As pre-pregnancy weight and BMI increased, both relative and absolute maternal weight gain during pregnancy decreased both in the study population as a whole (Supplement 6) and in the subgroup of healthy parturients (data not presented). A similar correlation was observed between pre-pregnancy BMI and the absolute increase in BMI by the end of pregnancy in both populations (Supplements 15 and 16, respectively).

## Discussion

Women who are overweight or obese pre-pregnancy have more chronic medical and obstetric comorbidities. Obese and morbidly obese women are far less likely to conceive or enter labor spontaneously, and they undergo CD more often than other women. This relatively high likelihood of CD is also seen in obese and morbidly obese primiparas which suggests it does not stem from having undergone prior CDs. Although obesity is viewed as a factor associated with a decreased likelihood of achieving successful labor with induction [16], we found no association between obesity and failed induction of labor, and the prevalence of CD due to failed induction did not differ across the BMI groups (regardless of parity). On the same note, a common indication for CD in morbidly obese primiparous and multiparous women in our cohort was suspected macrosomia. Yet the actual mean neonatal birth weight in the morbidly obese population was < 4.0 kg and lower than the birth weight in lower BMI groups in both groups. Finally, these women are more likely to have adverse pregnancy outcomes than their normal weight counterparts.

Our systematic review of prior literature (Supplement 1) revealed clinically meaningful heterogeneity in the time points at BMI determination and in BMI classifications; some studies refer to pre-pregnancy BMI [9, 17, 18], some to BMI during pregnancy [19, 20], and some to pre-delivery BMI [21, 22]. Some even used overlapping BMI classifications measured during different trimesters [6, 23, 24]. Even meta-analyses that have linked obesity with chronic medical morbidities and various obstetric outcomes have pooled studies using a variety of definitions [17, 23, 24]. This weighs heavily on current ability to draw clinically consistent and meaningful conclusions.

Our study has several strengths. It is one of the largest cohorts investigating pre-pregnancy and pre-delivery BMI using consistent and validated BMI classifications. In order to further refine our findings, we subdivided some of the traditional BMI groups, as recent data link lower than traditionally accepted BMI values with early development of comorbidities [25, 26]. Most studies investigating overweight and obese obstetric populations have focused on pregnancy-related comorbidities and obstetric outcomes, while chronic medical comorbidities usually remain unstudied or are mentioned in passing [6, 8, 18, 19, 27, 28]. We studied the prevalence of chronic medical conditions as well as obstetric comorbidities and delivery outcomes.

Our finding that pre-pregnancy obesity/morbid obesity is associated with a greater burden of chronic medical comorbidities and more obstetric comorbidities compared to normal weight parturients corresponds with the existing handful of preceding reports (Supplement 1) and systematic reviews [23, 29]. We found that women with pre-pregnancy morbid obesity are less likely to conceive spontaneously when compared to women with a normal BMI. In this too, our findings agree with prior studies. Dağ et al. suggested that the fertility of obese women might be impaired, [30] while others showed that the probability of pregnancy is reduced by 5% per unit of BMI exceeding 29 kg/m^2^ [31].

The proportion of women with a prior CD was higher among parturients with pre-pregnancy obesity and morbid obesity than among parturients with a normal pre-pregnancy weight. This could be related to BMI but also to increasing parity and age which occur in parallel to increasing BMI [32]. With increasing BMI, spontaneous onset of labor became less prevalent, whereas induction of labor and CD with no trial of labor became more prevalent. This finding was observed only in term deliveries and was observed in association with both higher pre-pregnancy and higher pre-delivery BMIs. Denison et al. showed that when compared with primiparous women with first trimester 20 ≤ BMI < 25 kg/m^2^, the OR for spontaneous onset of labor for women at term pregnancy on their first delivery decreased significantly with increasing BMI [6]. A Danish study also showed an increased risk for labor induction and CD in pre-pregnancy obese (*OR* 2.2, 95% *CI* 1.7–2.8) vs normal weight (*OR* 1.6, 95% *CI* 1.3–2.1) parturients [7].

Overweight, obese, and morbidly obese women had a decreasing likelihood of vaginal delivery and an increasing likelihood of CD when compared to normal weight parturients in our cohort. Recent guidelines regarding obesity in pregnancy report a higher prevalence of CD in this population [5, 33]. Several meta-analyses also noted that the likelihood of CD increases with increasing BMI [24, 34]. We sought to understand whether the higher likelihood of CD in this population is related to parity or prior CDs by studying a subgroup of primiparous women. Similar to prior studies of primiparous women [9, 35], we found that primiparous women with pre-pregnancy obesity and morbid obesity were more likely to undergo CD than primiparous women with a normal pre-pregnancy BMI.

Our rates of overweight, obesity, and morbid obesity were somewhat different than those described in at least one meta-analysis [36]. However, 71% of the cases included in the meta-analysis were from the USA, where the prevalence of obesity is highest among developed countries [3]. The proportion of women with pregestational DM and HTN in our study is higher than previously described in the obstetric population [37, 38]. This may have been caused by selection bias; women who had data on BMI and were therefore included in our analysis also had a higher rate of these chronic comorbidities than those who did not have data on BMI. The rates of gestational DM in our study were higher than those described by the American College of Gynecologists [39] but are well within the wide range of prevalence described in global estimates (< 1–28%) [40]. Lastly, we found no association between pre-pregnancy BMI and postpartum hemorrhage, which is a topic with conflicting evidence in the literature [18, 19].This study has several limitations. It was conducted in a single medical center. However, many of our findings are similar to those of prior publications, suggesting they may be generalizable nonetheless. We present no data on whether neuraxial analgesia was administered or not. Neuraxial analgesia may have influenced labor and postpartum complications as well as maternal and fetal outcomes [41–43]. We did not seek adjusted associations in this descriptive paper, and this should be performed in future research. Our study bears all the limitations of retrospective data analyses. We addressed documentation bias by comparing women with and without data on pre-pregnancy BMI, and the proportion of missing data in included cases was overall very low. Causation cannot be implied, but subgroup analysis on primiparas enables exclusion of high parity order or prior CD as the variables predominantly determining current CD. We chose to include only women up to 50 years of age. Older women constituted only a small proportion of our study population (> 40 years: 4.1% and > 45 years: 0.4%); therefore, additional age-related effects are highly unlikely.

## Conclusion

Pre-pregnancy obesity/morbid obesity is associated with lower rates of spontaneous conception, higher rates of chronic medical and obstetric comorbidities during pregnancy, lower rates of spontaneous labor, and higher rates of CD and adverse delivery outcomes. Whether these associations are caused by obesity or treatment remains unclear.

## Supplementary Information

**Additional file 1: Supplementary Table 1.** Comparison of chronic medical and obstetric comorbidities and pregnancy outcomes in previous studies. **Supplementary Fig. 2.** Flow chart illustrating the total number of cases that met research criteria. **Supplementary Table 3**. Comparison of selected variables between study population and cases excluded from study. **Supplementary Table 4.** Missing data in the study population by pre-pregnancy BMI. **Supplementary Table 5.** Age distribution of women admitted for delivery by pre-pregnancy BMI. **Supplementary Table 6. **Maternal age, height, weight and BMI at the beginning and end of pregnancy by pre-pregnancy BMI. **Supplementary Table 7.** Onset of labor, stratified by gestational age, by pre-pregnancy BMI. **Supplementary Fig. 8.** Odds ratio and 95% confidence interval for induction of labor by pre-pregnancy BMI. **Supplementary Fig. 9.** Odds ratio and 95% confidence interval for cesarean delivery with no trial of labor by pre-pregnancy BMI. **Supplementary Table 10.** Onset of labor, stratified by gestational age, by BMI at the end of pregnancy. **Supplementary Fig. 11.** Odds ratio and 95% confidence interval for induction of labor by end of pregnancy BMI. **Supplementary Fig. 12.** Odds ratio and 95% confidence interval for cesarean delivery with no trial of labor by end of pregnancy BMI. **Supplementary Table 13.** Indications for cesarean delivery in primiparous women by pre-pregnancy BMI. **Supplementary Table 14.** Distribution of the population as a whole and of the subgroup of healthy parturients into BMI groups based on pre-pregnancy BMI and BMI at the end of pregnancy. **Supplementary Fig. 15.** Correlation between pre-pregnancy BMI and increase in BMI during pregnancy in the study population. **Supplementary Fig. 16.** Correlation between pre-pregnancy BMI and increase in BMI during pregnancy in healthy parturients.

## Data Availability

The datasets used and/or analyzed during the current study are available from the corresponding author on reasonable request.
