# Supplementary material for: Medical and obstetric comorbidities and delivery outcomes in overweight and obese parturients: a retrospective analysis
Source: J Anesth Analg Crit Care. 2023 Jun 30;3:21. doi: 10.1186/s44158-023-00105-7 (PMC10311820; doi:10.1186/s44158-023-00105-7)
Supplement: Supplementary file 1 — Additional file 1: Supplementary Table 1. Comparison of chronic medical and obstetric comorbidities and pregnancy outcomes in previous studies. Supplementary Fig. 2. Flow chart illustrating the total number of cases that met research criteria. Supplementary Table 3. Comparison of selected variables between study population and cases excluded from study. Supplementary Table 4. Missing data in the study population by pre-pregnancy BMI. Supplementary Table 5. Age distribution of women admitted for delivery by pre-pregnancy BMI. Supplementary Table 6. Maternal age, height, weight and BMI at the beginning and end of pregnancy by pre-pregnancy BMI. Supplementary Table 7. Onset of labor, stratified by gestational age, by pre-pregnancy BMI. Supplementary Fig. 8. Odds ratio and 95% confidence interval for induction of labor by pre-pregnancy BMI. Supplementary Fig. 9. Odds ratio and 95% confidence interval for cesarean delivery with no trial of labor by pre-pregnancy BMI. Supplementary Table 10. Onset of labor, stratified by gestational age, by BMI at the end of pregnancy. Supplementary Fig. 11. Odds ratio and 95% confidence interval for induction of labor by end of pregnancy BMI. Supplementary Fig. 12. Odds ratio and 95% confidence interval for cesarean delivery with no trial of labor by end of pregnancy BMI. Supplementary Table 13. Indications for cesarean delivery in primiparous women by pre-pregnancy BMI. Supplementary Table 14. Distribution of the population as a whole and of the subgroup of healthy parturients into BMI groups based on pre-pregnancy BMI and BMI at the end of pregnancy. Supplementary Fig. 15. Correlation between pre-pregnancy BMI and increase in BMI during pregnancy in the study population. Supplementary Fig. 16. Correlation between pre-pregnancy BMI and increase in BMI during pregnancy in healthy parturients. [file 44158_2023_105_MOESM1_ESM.doc]

**Supplementary Tables and Figures**

Supplementary Table 1: Comparison of chronic medical and obstetric comorbidities and pregnancy outcomes in previous studies.

Supplementary Figure 2: Flow chart illustrating the total number of cases that met research criteria.

Supplementary Table 3: Comparison of selected variables between study population and cases excluded from study.

Supplementary Table 4: Missing data in the study population by pre-pregnancy BMI.

Supplementary Table 5: Age distribution of women admitted for delivery by pre-pregnancy BMI.

Supplementary Table 6: Maternal age, height, weight and BMI at the beginning and end of pregnancy by pre-pregnancy BMI.

Supplementary Table 7: Onset of labor, stratified by gestational age, by pre-pregnancy BMI.

Supplementary Figure 8: Odds ratio and 95% confidence interval for induction of labor by pre-pregnancy BMI.

Supplementary Figure 9: Odds ratio and 95% confidence interval for cesarean delivery with no trial of labor by pre-pregnancy BMI.

Supplementary Table 10: Onset of labor, stratified by gestational age, by BMI at the end of pregnancy.

Supplementary Figure 11: Odds ratio and 95% confidence interval for induction of labor by end of pregnancy BMI

Supplementary Figure 12: Odds ratio and 95% confidence interval for cesarean delivery with no trial of labor by end of pregnancy BMI.

Supplementary Table 13: Indications for cesarean delivery in primiparous women by pre-pregnancy BMI.

Supplementary Table 14: Distribution of the population as a whole and of the subgroup of healthy parturients into BMI groups based on pre-pregnancy BMI and BMI at the end of pregnancy.

Supplementary Figure 15: Correlation between pre-pregnancy BMI and increase in BMI during pregnancy in the study population.

Supplementary Figure 16: Correlation between pre-pregnancy BMI and increase in BMI during pregnancy in healthy parturients.

**Supplementary Table 1: Comparison of chronic medical and obstetric comorbidities and pregnancy outcomes in previous studies.**

| **Comorbidity/outcome**  **Study BMI group** | | **Pre-gestational DM** | | **Pre-gestational HTN** | | **Gestational DM** | | **Gestational HTN** | | **Pre-eclampsia** | | **Eclampsia** | | **Induction of labor** | | **Cesarean delivery** | | **PPH** | | **VTE** | |
| --- | --- | --- | --- | --- | --- | --- | --- | --- | --- | --- | --- | --- | --- | --- | --- | --- | --- | --- | --- | --- | --- |
|  |  | **%** | **OR (95%CI)** | **%** | **OR (95%CI)** | **%** | **OR  (95%CI)** | **%** | **OR (95%CI)** | **%** | **OR (95%CI)** | **%** | **OR (95%CI)** | **%** | **OR (95%CI)** | **%** | **OR (95%CI)** | **%** | **OR (95%CI)** | **%** | **OR (95%CI)** |
| **Metsälä ^28^, ^A^** | **Normal weight ^D^** | 1% | - | 1% | - | 5% ^#^ | 1  (ref) | 5% | 1  (ref) | 3% @ | 1  (ref) | <1% | 1  (ref) | - | - | - | - | - | - | - | - |
|  |  |  |  |  |  | 1% ^$^ |  |  |  | 1% ^&^ |  |  |  |  |  |  |  |  |  |  |  |
|  | **Overweight ^E^** | 2% | - | 2% | - | 16% ^#^ | 3.6  (3.4-3.8) ^#^ | 8% | 1.7  (1.6-1.8) | 4% @ | 1.5  (1.3-1.6) @ | <1% | 0.9  (0.4-1.8) | - | - | - | - | - | - | - | - |
|  |  |  |  |  |  | 2%^$^ | 3.0  (2.5-3.5) ^$^ |  |  | 1% ^&^ | 1.7 (1.4-2.0) ^&^ |  |  |  |  |  |  |  |  |  |  |
|  | **Obesity class I ^F^** | 3% | - | 5% | - | 25% ^#^ | 6.4  (6.0-6.9) ^#^ | 13% | 2.9  (2.7-3.2) | 7% @ | 2.3  (2.1-2.6) @ | <1% | 0.6  (0.2-2.6) | - | - | - | - | - | - | - | - |
|  |  |  |  |  |  | 5% ^$^ | 7.4  (6.3-8.8) ^$^ |  |  | 2% ^&^ | 2.3  (1.8-2.8) ^&^ |  |  |  |  |  |  |  |  |  |  |
|  | **Obesity class II ^G^** | 4% | - | 8% | - | 34% ^#^ | 9.7  (8.7-10.8) ^#^ | 16% | 3.8  (3.4-4.4) | 8% @ | 3.2  (2.6-3.8) @ | <1% | 1.8  (0.4-7.8) | - | - | - | - | - | - | - | - |
|  |  |  |  |  |  | 9% ^$^ | 12.3  (10.0-15.1) ^$^ |  |  | 2% ^&^ | 2.7  (2-3.8) ^&^ |  |  |  |  |  |  |  |  |  |  |
|  | **Obesity class III ^H^** | 7% | - | 11% | - | 42% ^#^ | 13.9  (11.9-16.2) ^#^ | 22% | 5.8  (4.9-6.9) | 9% @ | 3.7  (2.9-4.9) @ | <1% | 2.5  (0.3-18.2) | - | - | - | - | - | - | - | - |
|  |  |  |  |  |  | 13% ^$^ | 17.6  (13.5-22.9) ^$^ |  |  | 2% ^&^ | 2.3  (1.3-4.0) ^&^ |  |  |  |  |  |  |  |  |  |  |
| **Sebire ^19, B^** | **Normal weight ^I^** | 0.3% | - | 3.8% | - | 0.75% | 1  (ref) | - | - | 0.7% | 1  (ref) | - | - | 15.3% | 1  (ref) | 7.8% ^Δ^ | 1  (ref) | 10.4% | 1  (ref) | 0% | 1  (ref) |
|  |  |  |  |  |  |  |  |  |  |  |  |  |  |  |  | 4.1% ^Ω^ |  |  |  |  |  |
|  | **Moderate obesity ^E^** | 0.6% | - | 6.6% | - | 1.7% | 1.7  (1.5-1.8) | - | - | 1.0% | 1.4  (1.3-1.6) | - | - | 19.2% | 1.3  (1.2-1.3) | 10.3% ^Δ^ | 1.3  (1.3-1.3) ^Δ^ | 13.2% | 1.2  (1.1-1.2) | 0.1% | 1.4  (0.9-2.2) |
|  |  |  |  |  |  |  |  |  |  |  |  |  |  |  |  | 5.6% ^Ω^ | 1.2  (1.1-1.3) ^Ω^ |  |  |  |  |
|  | **Severe obesity ^J^** | 0.8% | - | 12.5% | - | 3.5% | 3.6  (3.3-4.0) | - | - | 1.4% | 2.1  (1.9-2.5) | - | - | 24.7% | 1.7  (1.6-1.8) | 13.4 ^Δ^ | 1.8  (1.7-1.9) ^Δ^ | 17.1% | 1.4  (1.3-1.5) | 0.1% | 1.5  (0.8-2.7) |
|  |  |  |  |  |  |  |  |  |  |  |  |  |  |  |  | 8.5% ^Ω^ | 1.7  (1.6-1.8) ^Ω^ |  |  |  |  |
| **Butwick ^18, A^** | **Normal weight ^D^** | 0.4% | - | 0.8% | - | - | - | 1.6% | - | 2.4%* | - | 2.4%* | - | 14.2% | - | 28.1% | - | 2.8% | 1  (ref) | - | - |
|  | **Overweight ^E^** | 0.9% | - | 1.8% | - | - | - | 2.4% | - | 3.2%* | - | 3.2%* | - | 15.3% | - | 34.0% | - | 2.9% | 1.1  (1.0-1.1) | - | - |
|  | **Obesity class I ^F^** | 1.7% | - | 3.3% | - | - | - | 3.3% | - | 4.2%* | - | 4.2%* | - | 16.4% | - | 39.6% | - | 2.8% | 1.1  (1.1-1.1) | - | - |
|  | **Obesity class II ^G^** | 2.7% | - | 5.6% | - | - | - | 4.3% | - | 5.1%* | - | 5.1%* | - | 17.9% | - | 45.5% | - | 2.6% | 1.0  (1.0-1.1) | - | - |
|  | **Obesity class III ^H^** | 4.2% | - | 9.8% | - | - | - | 5.3% | - | 6.0%* | - | 6.0%* | - | 18.7% | - | 53.7% | - | 2.6% | 1.0  (1.0-1.1) | - | - |
| **Chen ^27, A^** | **Normal weight ^K^** | - | - | - | - | 7.1% | - | 2.3% | - | 4.9% | - | - | - | - | - | 53.6% | - | - | - | - | - |
|  | **Overweight ^L^** | - | - | - | - | 14.9% | - | 6.3% | - | 9.6% | - | - | - | - | - | 65.0% | - | - | - | - | - |
|  | **Obesity ^M^** | - | - | - | - | 23.7% | - | 12.3% | - | 17.6% | - | - | - | - | - | 77.1% | - | - | - | - | - |
| **Denison ^6, C^** | **Normal weight ^I^** | - | - | - | 1  (ref) | - | 1  (ref) | - | 1  (ref) | - | - | - | - | - | - | - | 1  (ref) | - | 1  (ref) | - | - |
|  | **Overweight ^E^** | - | - | - | 2.3  (1.8-2.9) | - | 1.9  (1.7-2.2) | - | 1.8  (1.7-1.9) | - | - | - | - | - | - | - | 1.5  (1.4-1.5) | - | 1.1  (1.1-1.2) | - | - |
|  | **Obesity ^F^** | - | - | - | 4.8  (3.6-6.5) | - | 3.0  (2.5-3.5) | - | 2.9  (2.7-3.1) | - | - | - | - | - | - | - | 1.9  (1.8-2.0) | - | 1.2  (1.1-1.3) | - | - |
|  | **Severe obesity ^N^** | - | - | - | 9.1  (6.5-12.8) | - | 5.6  (4.6-6.8) | - | 4.2  (3.8-4.7) | - | - | - | - | - | - | - | 2.4  (2.2-2.6) | - | 1.4  (1.2-1.6) | - | - |
| **Hermann- primiparas ^9, A^** | **Normal weight ^D^** | 0.2% | - | 0.5% | - | 1.2% ^$^ | - | - | - | 2.2% | - | - | - | 23.8% | - | 20.9% | - | - | - | - | - |
|  | **Overweight ^E^** | 0.6% | - | 0.9% | - | 2.1% ^$^ | - | - | - | 3.6% | - | - | - | 31.0% | - | 25.9% | - | - | - | - | - |
|  | **Obesity ^J^** | 1.1% | - | 5.2% | - | 7.2% ^$^ | - | - | - | 7.0% | - | - | - | 42.6% | - | 34.6% | - | - | - | - | - |
| **Hermann- multiparas  ^9, A^** | **Normal weight ^D^** | 0.3% | - | 1.8% | - | 1.2% ^$^ | - | - | - | 0.9% | - | - | - | 18.6% | - | 15.7% | - | - | - | - | - |
|  | **Overweight ^E^** | 0.9% | - | 3.6% | - | 3.0% ^$^ | - | - | - | 2.2% | - | - | - | 22.7% | - | 20.7% | - | - | - | - | - |
|  | **Obesity ^J^** | 1.7% | - | 8.8% | - | 7.0% ^$^ | - | - | - | 4.4% | - | - | - | 23.6% | - | 29.5% | - | - | - | - | - |
| **Fuchs- French cohort ^8, B^** | **Normal weight ^D^** | 1.2% | - | 1.1% | - | - | - | - | - | 2.7%* | 1  (ref)* | 2.7%* | 1  (ref)* | - | - | 21.4% | 1  (ref) | - | - | 0.2% | 1  (ref) |
|  | **Overweight ^E^** | 1.7% | - | 2.7% | - | - | - | - | - | 3.6%* | 1.3  (1.2-1.5)* | 3.6%* | 1.3  (1.2-1.5)* | - | - | 27.5% | 1.2  (1.1-1.2) | - | - | 0.2% | 1.1  (0.6-1.8) |
|  | **Obesity class I ^F^** | 2.8% | - | 4.0% | - | - | - | - | - | 5.1%* | 2.1  (1.7-2.4)* | 5.1%* | 2.1  (1.7-2.4)* | - | - | 34.4% | 1.2  (1.2-1.3) | - | - | 0.2% | 1.5  (0.8-2.8) |
|  | **Obesity class II ^G^** | 4.2% | - | 6.6% | - | - | - | - | - | 8.1%* | 2.3  (1.8-2.9)* | 8.1%* | 2.3  (1.8-2.9)* | - | - | 39.8% | 1.3  (1.2-1.4) | - | - | 0.4% | 0.9  (0.3-2.6) |
|  | **Obesity class III ^H^** | 5.6% | - | 11.1% | - | - | - | - | - | 9.5%* | 2.8  (2.1-3.6)* | 9.5%* | 2.8  (2.1-3.6)* | - | - | 44.4% | 1.5  (1.3-1.6) | - | - | 0% | 0.9  (0.2-3.8) |
| **Fuchs- Canadian cohort ^8, B^** | **Normal weight ^D^** | 0.6% | - | 0.8% | - | - | - | - | - | 2.1%* | - | 2.1%* | - | - | - | 19.2% | - | - | - | 0.2% | - |
|  | **Overweight ^E^** | 1.3% | - | 1.5% | - | - | - | - | - | 3.4%* | - | 3.4%* | - | - | - | 25.5% | - | - | - | 0.1% | - |
|  | **Obesity class I ^F^** | 2.4% | - | 3.3% | - | - | - | - | - | 5.7%* | - | 5.7%* | - | - | - | 28.8% | - | - | - | 0.4% | - |
|  | **Obesity class II ^G^** | 2.1% | - | 6.0% | - | - | - | - | - | 7.1%* | - | 7.1%* | - | - | - | 32.3% | - | - | - | 0.2% | - |
|  | **Obesity class III ^H^** | 4.6% | - | 10.5% | - | - | - | - | - | 10.5%* | - | 10.5%* | - | - | - | 38.4% | - | - | - | 0.4% | - |

**search strategy and data extraction for comparison to previous studies:** An online search was conducted for relevant publications in PubMed, Embase and Web of Science. The search was restricted to articles written in English published between January 1950 and September 2021. We sought observational studies, randomized controlled trials, case–control, longitudinal and cross-sectional studies that reported the prevalence of chronic and obstetric maternal morbidity in relation to different pre-pregnancy BMI groups of women admitted for delivery. The main journals most likely to contain publications in this area were also identified using content experts in the area and hand searched. Abstracts from the selected articles were read, and if considered eligible for further review by the authors (RG and SE), the complete article was obtained for screening of content and hand searching of reference lists. The data from articles containing information relevant to the current study were tabulated.
DM- diabetes mellitus; HTN- hypertension; PPH- postpartum hemorrhage; VTE- venous thromboembolism; %- percent; OR- odds ratio; CI- confidence interval; BMI- body mass index; kg- kilogram; m- meter; ref- reference.
**A**- BMI was measured pre-pregnancy or during first trimester; **B**- BMI was measured during pregnancy; **C**- BMI was measured during first and third trimesters; **D**- 18.5≤BMI<25 kg/m^2^; **E**- 25≤BMI<30 kg/m^2^; **F**- 30≤BMI<35 kg/m^2^; **G**- 35≤BMI<40 kg/m^2^; **H**- BMI≥40 kg/m^2^;**I**- 20≤BMI<25 kg/m^2^; **J**- BMI≥30 kg/m^2^; **K**- 18.5≤BMI<23 kg/m^2^; **L**- 23≤BMI<27.5 kg/m^2^; **M**- BMI≥27.5 kg/m^2^; **N**- BMI≥35 kg/m^2^; *- a composite variable of preeclampsia and eclampsia; @- mild to moderate preeclampsia; &- severe preeclampsia; #- not requiring insulin treatment; $- requiring insulin treatment.

**Supplementary Figure 2:** Flow chart illustrating the total number of cases that met research criteria.


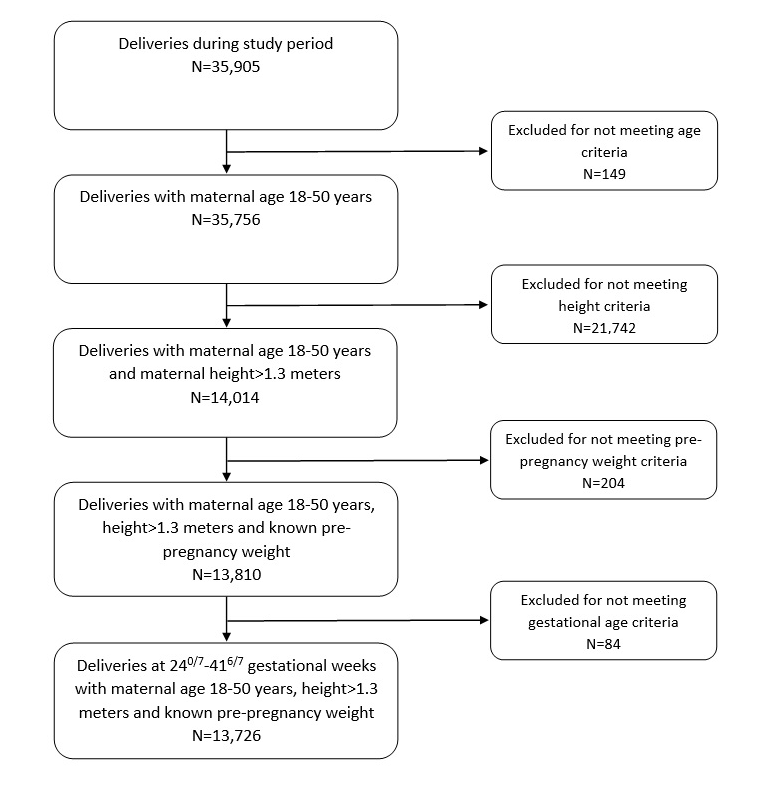


**Supplementary Table 3:** Comparison of selected variables between study population and cases excluded from study.

|  | **Study population  (N=13,726)** | | | **Cases excluded from study  (N=22,179)** | | |
| --- | --- | --- | --- | --- | --- | --- |
|  | **Mean ± SD** | **N (%)** | **CI [%]** | **Mean ± SD** | **N (%)** | **CI [%]** |
| **Age (y)** | 31.40±5.16 | - | - | 31.43±5.32 | - | - |
| **Height (m)** | 1.63±0.06 | - | - | 1.63±0.07 | - | - |
| **Smoking** | - | 784 (5.71%) | 5.32-6.10 | - | 1,239 (5.59%) | 5.27-5.91 |
| **Alcohol consumption** | - | 42  (0.31%) | 0.21-0.40 | - | 49  (0.22%) | 0.15-0.29 |
| **Proportion of healthy parturients** | - | 11,247 (81.94%) | 81.30-82.58 | - | 19,253 (86.81%) | 86.36-87.25 |
| **Pre-gestational DM (Type 1 or 2)** | - | 851 (6.20%) | 5.80-6.60 | - | 920  (4.15%) | 3.89-4.41 |
| **Pre-gestational HTN** | - | 616 (4.49%) | 4.14-4.83 | - | 742 (3.35%) | 3.11-3.58 |
| **Parity (number of delivery)** | 2.14±1.28 | - | - | 2.35±1.38 | - | - |
| **Past CD** | - | 1,964 (14.31%) | 13.72-14.89 | - | 2,956 (13.33%) | 12.88-13.78 |
| **Method of conception- spontaneous** | - | 11,475 (90.79%) | 90.29-91.29 | - | 18,235 (92.67%) | 92.30-93.03 |
| **Singleton pregnancy** | - | 13,283 (96.83%) | 96.54-97.12 | - | 21,617 (97.59%) | 97.39-97.79 |
| **Gestational DM** | - | 1,548 (11.28%) | 10.75-11.81 | - | 1,652 (7.45%) | 7.10-7.79 |
| **Gestational HTN** | - | 612 (4.46%) | 4.11-4.80 | - | 764 (3.44%) | 3.20-3.68 |
| **Preeclampsia/Eclampsia** | - | 435 (3.17%) | 2.88-3.46 | - | 557 (2.51%) | 2.31-2.72 |
| **Intrauterine growth restriction** | - | 415 (3.02%) | 2.74-3.31 | - | 495 (2.23%) | 2.04-2.43 |
| **Gestational age at delivery (wk)** | 39^0/7^±13d | - | - | 39^0/7^±16d | - | - |
| **Mode of delivery- CD** | - | 3,207 (23.37%) | 22.66-24.08 | - | 4,252 (19.17%) | 18.65-19.69 |
| **ICU admission** | - | 76  (0.55%) | 0.43-0.68 | - | 93  (0.42%) | 0.33-0.50 |

N- number; SD- standard deviation; y- year; m- meter; DM- diabetes mellitus; HTN- hypertension; CD- cesarean
delivery; wk- week; d- day; ICU- intensive care unit.

**Supplementary Table 4: Missing data in the study population by pre-pregnancy BMI.**

|  | **Pre-Pregnancy BMI** | | | | | | | | | | | | | |  | |
| --- | --- | --- | --- | --- | --- | --- | --- | --- | --- | --- | --- | --- | --- | --- | --- | --- |
|  | **Underweight (N=1,074)** | | **Normal Weight 1 (N=5,473)** | | **Normal Weight 2 (N=2,957)** | | **Overweight 1 (N=1,734)** | | **Overweight 2 (N=990)** | | **Obesity  (N=1,039)** | | **Morbid Obesity (N=459)** | | **Total  (N=13,726)** | |
|  | **N** | **%** | **N** | **%** | **N** | **%** | **N** | **%** | **N** | **%** | **N** | **%** | **N** | **%** | **N** | **%** |
| **Age** | 0 | 0% | 0 | 0% | 0 | 0% | 0 | 0% | 0 | 0% | 0 | 0% | 0 | 0% | 0 | 0% |
| **Height** | 0 | 0% | 0 | 0% | 0 | 0% | 0 | 0% | 0 | 0% | 0 | 0% | 0 | 0% | 0 | 0% |
| **Pre-pregnancy BMI** | 0 | 0% | 0 | 0% | 0 | 0% | 0 | 0% | 0 | 0% | 0 | 0% | 0 | 0% | 0 | 0% |
| **Gestational weight gain** | 90 | 8.40% | 582 | 10.63% | 364 | 12.31% | 219 | 12.63% | 115 | 11.62% | 126 | 12.13% | 65 | 14.16% | 1,561 | 11.37% |
| **Increase in BMI during pregnancy** | 90 | 8.40% | 582 | 10.63% | 364 | 12.31% | 219 | 12.63% | 115 | 11.62% | 126 | 12.13% | 65 | 14.16% | 1,561 | 11.37% |
| **BMI at end of index pregnancy** | 90 | 8.40% | 582 | 10.63% | 364 | 12.31% | 219 | 12.63% | 115 | 11.62% | 126 | 12.13% | 65 | 14.16% | 1,561 | 11.37% |
| **Smoking** | 50 | 4.66% | 206 | 3.76% | 108 | 3.65% | 80 | 4.61% | 40 | 4.04% | 52 | 5.00% | 20 | 4.36% | 556 | 4.05% |
| **Alcohol consumption** | 57 | 5.31% | 259 | 4.73% | 128 | 4.33% | 96 | 5.54% | 44 | 4.44% | 51 | 4.91% | 25 | 5.45% | 660 | 4.81% |
| **Drug abuse** | 73 | 6.80% | 348 | 6.36% | 160 | 5.41% | 127 | 7.32% | 53 | 5.35% | 66 | 6.35% | 32 | 6.97% | 859 | 6.26% |
| **Pre-gestational DM (Type 1 or 2)** | 0 | 0% | 0 | 0% | 0 | 0% | 0 | 0% | 0 | 0% | 0 | 0% | 0 | 0% | 0 | 0% |
| **Pre-gestational HTN** | 0 | 0% | 0 | 0% | 0 | 0% | 0 | 0% | 0 | 0% | 0 | 0% | 0 | 0% | 0 | 0% |
| **Parity** | 0 | 0% | 0 | 0% | 0 | 0% | 0 | 0% | 0 | 0% | 0 | 0% | 0 | 0% | 0 | 0% |
| **Past cesarean delivery** | 0 | 0% | 0 | 0% | 0 | 0% | 0 | 0% | 0 | 0% | 0 | 0% | 0 | 0% | 0 | 0% |
| **Method of conception** | 81 | 7.54% | 435 | 7.95% | 231 | 7.81% | 152 | 8.77% | 81 | 8.18% | 73 | 7.03% | 36 | 7.84% | 1,089 | 7.93% |
| **Number of fetuses** | 1 | 0.09% | 3 | 0.05% | 1 | 0.03% | 3 | 0.17% | 0 | 0% | 0 | 0% | 0 | 0% | 8 | 0.06% |
| **Gestational DM** | 0 | 0% | 0 | 0% | 0 | 0% | 0 | 0% | 0 | 0% | 0 | 0% | 0 | 0% | 0 | 0% |
| **Gestational HTN** | 0 | 0% | 0 | 0% | 0 | 0% | 0 | 0% | 0 | 0% | 0 | 0% | 0 | 0% | 0 | 0% |
| **Preeclampsia/ Eclampsia** | 0 | 0% | 0 | 0% | 0 | 0% | 0 | 0% | 0 | 0% | 0 | 0% | 0 | 0% | 0 | 0% |
| **Intrauterine growth restriction** | 0 | 0% | 0 | 0% | 0 | 0% | 0 | 0% | 0 | 0% | 0 | 0% | 0 | 0% | 0 | 0% |
| **Onset of labor** | 51 | 4.75% | 283 | 5.17% | 149 | 5.04% | 124 | 7.15% | 57 | 5.76% | 76 | 7.31% | 34 | 7.41% | 774 | 5.64% |
| **Mode of delivery** | 0 | 0% | 3 | 0.05% | 0 | 0% | 0 | 0% | 0 | 0% | 0 | 0% | 1 | 0.22% | 4 | 0.03% |
| **Urgency of cesarean delivery** | 1 | 0.52% | 5 | 0.48% | 2 | 0.31% | 2 | 0.43% | 4 | 1.36% | 2 | 0.58% | 2 | 0.97% | 18 | 0.13% |
| **Post-partum hemorrhage** | 0 | 0% | 0 | 0% | 0 | 0% | 0 | 0% | 0 | 0% | 0 | 0% | 0 | 0% | 0 | 0% |
| **ICU admission** | 0 | 0% | 0 | 0% | 0 | 0% | 0 | 0% | 0 | 0% | 0 | 0% | 0 | 0% | 0 | 0% |

BMI- body mass index; N- number; %- percentage; DM- diabetes mellitus; HTN- hypertension; Preeclampsia/eclampsia (any)- a composite variable of preeclampsia, superimposed preeclampsia, HELLP (hemolysis, elevated liver enzymes and low platelets) syndrome and eclampsia; ICU- intensive care unit.

**Supplementary Table 5: Age distribution of women admitted for delivery by pre-pregnancy BMI.**

| **Age** | **Pre-Pregnancy BMI** | | | | | | | | | | | | | |
| --- | --- | --- | --- | --- | --- | --- | --- | --- | --- | --- | --- | --- | --- | --- |
|  | **Underweight (N=1,074)** | | **Normal Weight 1 (N=5,473)** | | **Normal Weight 2 (N=2,957)** | | **Overweight 1 (N=1,734)** | | **Overweight 2 (N=990)** | | **Obesity  (N=1,039)** | | **Morbid Obesity (N=459)** | |
|  | **N (%)** | **CI [%]** | **N (%)** | **CI [%]** | **N (%)** | **CI [%]** | **N (%)** | **CI [%]** | **N (%)** | **CI [%]** | **N (%)** | **CI [%]** | **N (%)** | **CI [%]** |
| **18 to <20 (y)** | 18 (1.68%) | 1.00-2.64^▲^ | 34 (0.62%) | 0.43-0.87 | 17 (0.57%) | 0.34-0.92 | 11 (0.63%) | 0.32-1.13 | 10 (1.01%) | 0.49-1.85 | 2  (0.19%) | 0.02-0.69 | 1  (0.22%) | 0.01-1.21 |
| **20 to <25 (y)** | 175 (16.29%) | 14.13-18.64^▲^ | 600 (10.96%) | 10.15-11.82 | 288 (9.74%) | 8.69-10.87 | 158 (9.11%) | 7.80-10.57 | 95 (9.60%) | 7.83-11.60 | 84 (8.08%) | 6.50-9.91^□^ | 28 (6.10%) | 4.09-8.70^□^ |
| **25 to <30 (y)** | 371 (34.54%) | 31.70-37.47 | 1,699 (31.04%) | 29.82-32.29 | 876 (29.62%) | 27.98-31.31 | 465 (26.82%) | 24.74-28.97^□^ | 243 (24.55%) | 21.89-27.35^■^ | 253 (24.35%) | 21.77-27.08^■^ | 119 (25.93%) | 21.97-30.19 |
| **30 to <35 (y)** | 338 (31.47%) | 28.70-34.34 | 1,948 (35.59%) | 34.32-36.88 | 1,094 (37.00%) | 35.25-38.77 | 608 (35.06%) | 32.82-37.36 | 334 (33.74%) | 30.79-36.78 | 328 (31.57%) | 28.75-34.49 | 161 (35.08%) | 30.71-39.64 |
| **35 to <40 (y)** | 142 (13.22%) | 11.25-15.39^■^ | 973 (17.78%) | 16.77-18.82 | 546 (18.46%) | 17.08-19.91 | 388 (22.38%) | 20.43-24.41^▲^ | 227 (22.93%) | 20.34-25.68^▲^ | 285 (27.43%) | 24.74-30.25^▲^ | 118 (25.71%) | 21.77-29.96^▲^ |
| **40 to <45 (y)** | 25 (2.33%) | 1.51-3.42 | 199 (3.64%) | 3.16-4.17 | 123 (4.16%) | 3.47-4.94 | 92 (5.31%) | 4.30-6.47^∆^ | 74 (7.47%) | 5.91-9.29^▲^ | 76 (7.31%) | 5.81-9.07^▲^ | 26 (5.66%) | 3.73-8.19 |
| **45 to ≤50 (y)** | 5  (0.47%) | 0.15-1.08 | 20 (0.37%) | 0.22-0.56 | 13 (0.44%) | 0.23-0.75 | 12 (0.69%) | 0.36-1.21 | 7  (0.71%) | 0.28-1.45 | 11 (1.06%) | 0.53-1.89 | 6  (1.31%) | 0.48-2.82 |

BMI- body mass index; N- number; CI- 95% confidence interval; y- year.
* Percentage (%) represent the proportion of cases out of the relevant pre-pregnancy BMI subgroup.
∆- higher than Normal weight 1; ▲- higher than Normal weight 1 and Normal weight 2; □- lower than Normal weight 1; ■- lower than Normal weight 1 and Normal weight 2.

**Supplementary Table 6: Maternal age, height, weight and BMI at the beginning and end of pregnancy by pre-pregnancy BMI.**

|  | **Pre-Pregnancy BMI** | | | | | | | | | | | | | |
| --- | --- | --- | --- | --- | --- | --- | --- | --- | --- | --- | --- | --- | --- | --- |
|  | **Underweight (N=1,074)** | | **Normal Weight 1 (N=5,473)** | | **Normal Weight 2 (N=2,957)** | | **Overweight 1 (N=1,734)** | | **Overweight 2 (N=990)** | | **Obesity  (N=1,039)** | | **Morbid Obesity (N=459)** | |
|  | **N (%)** | **Mean ± SD** | **N (%)** | **Mean ± SD** | **N (%)** | **Mean ± SD** | **N (%)** | **Mean ± SD** | **N (%)** | **Mean ± SD** | **N (%)** | **Mean ± SD** | **N (%)** | **Mean ± SD** |
| **Age (y)** | 1,074 (100%) | 29.8±5.1 | 5,473 (100%) | 31.1±5.0 | 2,957 (100%) | 31.4±5.0 | 1,734 (100%) | 31.9±5.2 | 990 (100%) | 32.2±5.5 | 1,039 (100%) | 32.6±5.5 | 459 (100%) | 32.7±5.1 |
| **Height (m)** | 1,074 (100%) | 1.64±0.06 | 5,473 (100%) | 1.63±0.06 | 2,957 (100%) | 1.63±0.06 | 1,734 (100%) | 1.63±0.06 | 990 (100%) | 1.63±0.06 | 1,039 (100%) | 1.63±0.06 | 459 (100%) | 1.63±0.06 |
| **Weight at the beginning of index pregnancy (kg)** | 1,074 (100%) | 47.0±4.1 | 5,473 (100%) | 55.0±5.1 | 2,957 (100%) | 63.0±5.2 | 1,734 (100%) | 69.1±5.7 | 990 (100%) | 75.8±6.2 | 1,039 (100%) | 85.3±7.6 | 459 (100%) | 102.6±10.9 |
| **Gestational Weight gain (kg)** | 984 (91.6%) | 12.7±4.9 | 4,891 (89.4%) | 13.0±5.0 | 2,593 (87.7%) | 12.8±5.5 | 1,515 (87.4%) | 12.1±6.0 | 875 (88.4%) | 10.7±6.5 | 913 (87.9%) | 8.6±6.9 | 394 (85.8%) | 6.1±7.1 |
| **Weight at end of index pregnancy (kg)** | 984 (91.6%) | 59.7±6.8 | 4,891 (89.4%) | 68.0±7.6 | 2,593 (87.7%) | 75.7±8.0 | 1,515 (87.4%) | 81.3±8.7 | 875 (88.4%) | 86.5±9.3 | 913 (87.9%) | 94.0±10.0 | 394 (85.8%) | 108.7±12.7 |
| **BMI at the beginning of index pregnancy (kg/m^2^)** | 1,074 (100%) | 17.6±0.8 | 5,473 (100%) | 20.6±1.1 | 2,957 (100%) | 23.7±0.7 | 1,734 (100%) | 26.1±0.7 | 990 (100%) | 28.6±0.7 | 1,039 (100%) | 32.0±1.4 | 459 (100%) | 38.5±3.1 |
| **Increase in BMI during pregnancy (kg/m^2^)** | 984 (91.6%) | 4.7±1.8 | 4,891 (89.4%) | 4.9±1.9 | 2,593 (87.7%) | 4.8±2.1 | 1,515 (87.4%) | 4.6±2.2 | 875 (88.4%) | 4.0±2.4 | 913 (87.9%) | 3.2±2.6 | 394 (85.8%) | 2.3±2.7 |
| **BMI at end of index pregnancy (kg/m^2^)** | 984 (91.6%) | 22.3±2.0 | 4,891 (89.4%) | 25.5±2.2 | 2,593 (87.7%) | 28.5±2.2 | 1,515 (87.4%) | 30.6±2.3 | 875 (88.4%) | 32.7±2.5 | 913 (87.9%) | 35.3±2.8 | 394 (85.8%) | 40.8±3.7 |

BMI- body mass index; N- number; SD- standard deviation; y- year; m- meter; kg- kilogram.

**Supplementary Table 7: Onset of labor, stratified by gestational age, by pre-pregnancy BMI.**

|  | | **Pre-Pregnancy BMI** | | | | | | | | | | | | | |
| --- | --- | --- | --- | --- | --- | --- | --- | --- | --- | --- | --- | --- | --- | --- | --- |
|  |  | **Underweight (N=1,074)** | | **Normal Weight 1 (N=5,473)** | | **Normal Weight 2 (N=2,957)** | | **Overweight 1 (N=1,734)** | | **Overweight 2 (N=990)** | | **Obesity (N=1,039)** | | **Morbid Obesity (N=459)** | |
|  |  | **N (%)** | **CI [%]** | **N (%)** | **CI [%]** | **N (%)** | **CI [%]** | **N (%)** | **CI [%]** | **N (%)** | **CI [%]** | **N (%)** | **CI [%]** | **N (%)** | **CI [%]** |
| **Gestational age** | **Onset of delivery** |  |  |  |  |  |  |  |  |  |  |  |  |  |  |
| **24^0/7^-33^6/7^ (wk)** | **Spontaneous onset of labor** | 15 (62.50%) | 40.59-81.20 | 32 (49.23%) | 36.60-61.93 | 25 (52.08%) | 37.19-66.71 | 5 (35.71%) | 12.76-64.86 | 6 (42.86%) | 17.66-71.14 | 2 (22.22%) | 2.81-60.01 | 2 (18.18%) | 2.28-51.78 |
|  | **Induction of labor** | 3 (12.50%) | 2.66-32.36 | 6 (9.23%) | 3.46-19.02 | 6 (12.50%) | 4.73-25.25 | 1  (7.14%) | 0.18-33.87 | 2 (14.29%) | 1.78-42.81 | 0  (0.00%) | 0.00-33.63 | 1  (9.09%) | 0.23-41.28 |
|  | **CD- No Trial of Labor** | 6 (25.00%) | 9.77-46.71 | 27 (41.54%) | 29.44-54.44 | 17 (35.42%) | 22.16-50.54 | 8 (57.14%) | 28.86-82.34 | 6 (42.86%) | 17.66-71.14 | 7 (77.78%) | 39.99-97.19 | 8 (72.73%) | 39.03-93.98 |
| **34^0/7^-36^6/7^ (wk)** | **Spontaneous onset of labor** | 30 (46.15%) | 33.70-58.97 | 114 (39.72%) | 34.02-45.64 | 56 (35.44%) | 28.01-43.44 | 37 (34.58%) | 25.65-44.39 | 25 (36.76%) | 25.39-49.33 | 25 (36.76%) | 25.39-49.33 | 9 (32.14%) | 15.88-52.35 |
|  | **Induction of labor** | 12 (18.46%) | 9.92-30.03 | 62 (21.60%) | 16.98-26.82 | 41 (25.95%) | 19.31-33.51 | 22 (20.56%) | 13.36-29.46 | 15 (22.06%) | 12.90-33.76 | 11 (16.18%) | 8.36-27.10 | 3 (10.71%) | 2.27-28.23 |
|  | **CD- No Trial of Labor** | 23 (35.38%) | 23.92-48.23 | 111 (38.68%) | 33.01-44.58 | 61 (38.61%) | 30.98-46.67 | 48 (44.86%) | 35.23-54.78 | 28 (41.18%) | 29.37-53.77 | 32 (47.06%) | 34.83-59.55 | 16 (57.14%) | 37.18-75.54 |
| **37^0/7^-41^6/7^ (wk)** | **Spontaneous onset of labor** | 589 (63.06%) | 59.88-66.17 | 3,007 (62.15%) | 60.77-63.52 | 1,474 (56.65%) | 54.72-58.56^□^ | 798 (53.59%) | 51.02-56.15^□^ | 382 (44.89%) | 41.51-48.30^■^ | 355 (40.07%) | 36.82-43.38^■^ | 122 (31.61%) | 27.00-36.50^■^ |
|  | **Induction of labor** | 257 (27.52%) | 24.67-30.50 | 1,312 (27.12%) | 25.87-28.40 | 798 (30.67%) | 28.90-32.48^∆^ | 462 (31.03%) | 28.68-33.45^∆^ | 311 (36.55%) | 33.30-39.88^▲^ | 346 (39.05%) | 35.82-42.35^▲^ | 156 (40.41%) | 35.48-45.50^▲^ |
|  | **CD- No Trial of Labor** | 88 (9.42%) | 7.63-11.48 | 519 (10.73%) | 9.87-11.63 | 330 (12.68%) | 11.43-14.02 | 229 (15.38%) | 13.58-17.31^∆^ | 158 (18.57%) | 16.01-21.34^▲^ | 185 (20.88%) | 18.25-23.71^▲^ | 108 (27.98%) | 23.56-32.74^▲^ |

BMI- body mass index; N- number; CI- 95% confidence interval; wk- week; CD- cesarean delivery.
∆- higher than Normal weight 1; ▲- higher than Normal weight 1 and Normal weight 2; □- lower than Normal weight 1; ■- lower than Normal weight 1 and Normal weight 2.

**Supplementary Figure 8**: Odds ratio and 95% confidence interval for induction of labor by pre-pregnancy BMI.


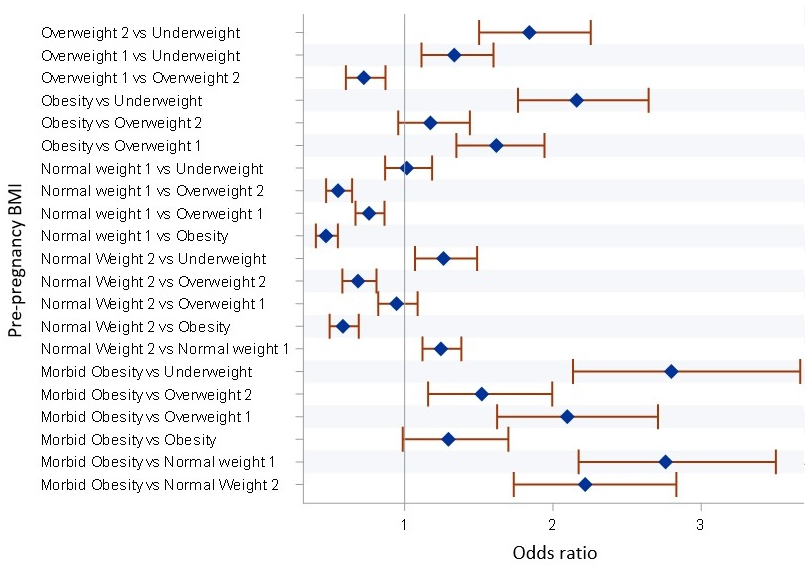


**Supplementary Figure 9**: Odds ratio and 95% confidence interval for cesarean delivery with no trial of labor by pre-pregnancy BMI.


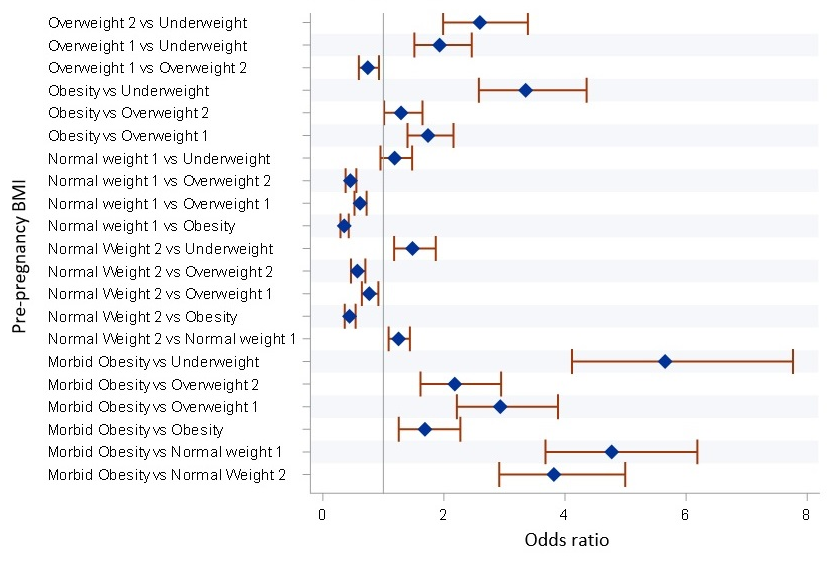


**Supplementary Table 10: Onset of labor, stratified by gestational age, by BMI at the end of pregnancy.**

|  | | **BMI at the end of pregnancy** | | | | | | | | | | | | | |
| --- | --- | --- | --- | --- | --- | --- | --- | --- | --- | --- | --- | --- | --- | --- | --- |
|  |  | **Underweight (N=28)** | | **Normal Weight 1 (N=903)** | | **Normal Weight 2 (N=2,186)** | | **Overweight 1 (N=2,918)** | | **Overweight 2 (N=2,449)** | | **Obesity  (N=2,604)** | | **Morbid Obesity (N=1,077)** | |
|  |  | **N (%)** | **CI [%]** | **N (%)** | **CI [%]** | **N (%)** | **CI [%]** | **N (%)** | **CI [%]** | **N (%)** | **CI [%]** | **N (%)** | **CI [%]** | **N (%)** | **CI [%]** |
| **Gestational age** | **Onset of delivery** |  |  |  |  |  |  |  |  |  |  |  |  |  |  |
| **24^0/7^-33^6/7^ (wk)** | **Spontaneous onset of labor** | 1 (100.0%) | 2.50-100.0 | 16 (51.61%) | 33.06-69.85 | 15 (46.88%) | 29.09-65.26 | 17 (51.52%) | 33.54-69.20 | 8 (36.36%) | 17.20-59.34 | 10 (40.00%) | 21.13-61.33 | 2 (18.18%) | 2.28-51.78 |
|  | **Induction of labor** | 0 (0.00%) | 0.00-97.50 | 6 (19.35%) | 7.45-37.47 | 1  (3.13%) | 0.08-16.22 | 3  (9.09%) | 1.92-24.33 | 2  (9.09%) | 1.12-29.16 | 3 (12.00%) | 2.55-31.22 | 1  (9.09%) | 0.23-41.28 |
|  | **CD- No Trial of Labor** | 0 (0.00%) | 0.00-97.50 | 9 (29.03%) | 14.22-48.04 | 16 (50.00%) | 31.89-68.11 | 13 (39.39%) | 22.91-57.86 | 12 (54.55%) | 32.21-75.61 | 12 (48.00%) | 27.80-68.69 | 8 (72.73%) | 39.03-93.98 |
| **34^0/7^-36^6/7^ (wk)** | **Spontaneous onset of labor** | 2 (50.00%) | 6.76-93.24 | 31 (44.93%) | 32.92-57.38 | 55 (44.72%) | 35.75-53.94 | 50 (35.71%) | 27.80-44.25 | 48 (36.92%) | 28.63-45.83 | 57 (37.50%) | 29.79-45.71 | 19 (27.54%) | 17.46-39.62 |
|  | **Induction of labor** | 0 (0.00%) | 0.00-60.24 | 15 (21.74%) | 12.71-33.31 | 26 (21.14%) | 14.30-29.42 | 33 (23.57%) | 16.81-31.48 | 36 (27.69%) | 20.21-36.22 | 29 (19.08%) | 13.17-26.24 | 14 (20.29%) | 11.56-31.69 |
|  | **CD- No Trial of Labor** | 2 (50.00%) | 6.76-93.24 | 23 (33.33%) | 22.44-45.71 | 42 (34.15%) | 25.84-43.24 | 57 (40.71%) | 32.50-49.33 | 46 (35.38%) | 27.20-44.25 | 66 (43.42%) | 35.41-51.69 | 36 (52.17%) | 39.80-64.35 |
| **37^0/7^-41^6/7^ (wk)** | **Spontaneous onset of labor** | 11 (50.00%) | 28.22-71.78 | 489 (65.03%) | 61.50-68.44 | 1,211 (62.94%) | 60.74-65.10 | 1,574 (60.31%) | 58.40-62.19 | 1,254 (57.81%) | 55.70-59.90^■^ | 1,136 (50.15%) | 48.07-52.23^■^ | 318 (34.53%) | 31.46-37.70^■^ |
|  | **Induction of labor** | 7 (31.82%) | 13.86-54.87 | 185 (24.60%) | 21.56-27.84 | 505 (26.25%) | 24.29-28.27 | 756 (28.97%) | 27.23-30.75 | 677 (31.21%) | 29.27-33.21^▲^ | 785 (34.66%) | 32.70-36.66^▲^ | 395 (42.89%) | 39.66-46.16^▲^ |
|  | **CD- No Trial of Labor** | 4 (18.18%) | 5.19-40.28 | 78 (10.37%) | 8.29-12.78 | 208 (10.81%) | 9.46-12.28 | 280 (10.73%) | 9.57-11.98 | 238 (10.97%) | 9.69-12.36 | 344 (15.19%) | 13.73-16.73^▲^ | 208 (22.58%) | 19.92-25.42^▲^ |

BMI- body mass index; N- number; CI- 95% confidence interval; wk- week; CD- cesarean delivery.
∆- higher than Normal weight 1; ▲- higher than Normal weight 1 and Normal weight 2; □- lower than Normal weight 1; ■- lower than Normal weight 1 and Normal weight 2.

**Supplementary Figure 11**: Odds ratio and 95% confidence interval for induction of labor by end of pregnancy BMI.


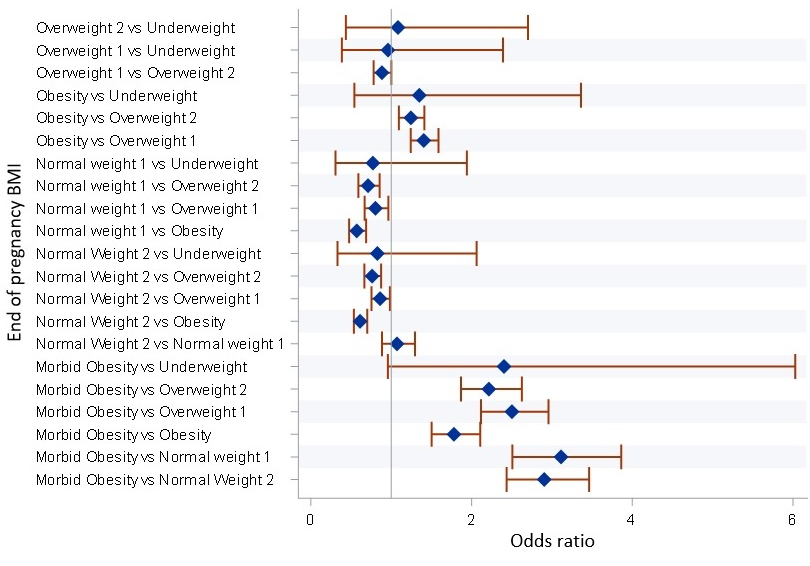


**Supplementary Figure 12**: Odds ratio and 95% confidence interval for cesarean delivery with no trial of labor by end of pregnancy BMI.


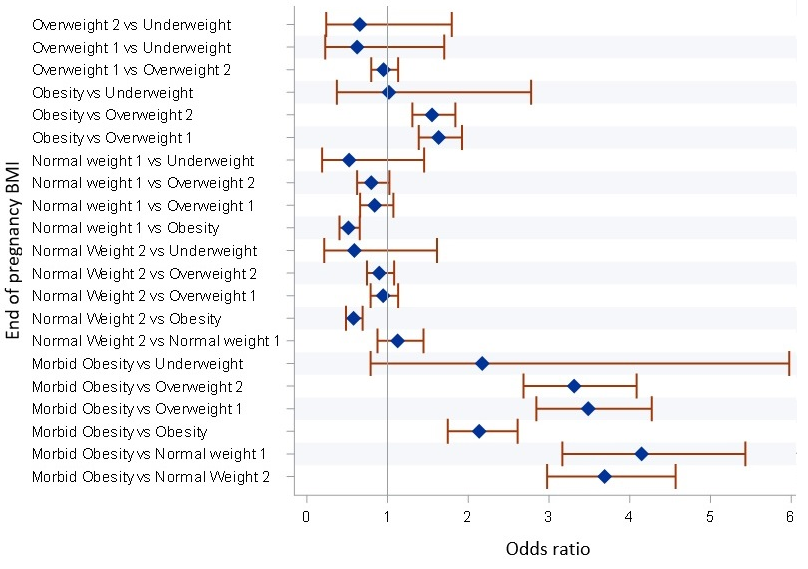


**Supplementary Table 13: Indications for cesarean delivery in primiparous women** **by pre-pregnancy BMI.**

|  | **Pre-Pregnancy BMI** | | | | | | | | | | | | | |
| --- | --- | --- | --- | --- | --- | --- | --- | --- | --- | --- | --- | --- | --- | --- |
|  | **Underweight (N=525)** | | **Normal Weight 1 (N=2,335)** | | **Normal Weight 2 (N=1,053)** | | **Overweight 1 (N=587)** | | **Overweight 2 (N=309)** | | **Obesity  (N=335)** | | **Morbid Obesity (N=157)** | |
|  | **N (%)** | **CI [%]** | **N (%)** | **CI [%]** | **N (%)** | **CI [%]** | **N (%)** | **CI [%]** | **N (%)** | **CI [%]** | **N (%)** | **CI [%]** | **N (%)** | **CI [%]** |
| **Dystocia** | 10 (11.24%) | 5.52-19.69 | 50 (11.55%) | 8.69-14.94 | 24 (9.84%) | 6.40-14.28 | 26 (15.12%) | 10.12-21.36 | 9 (10.23%) | 4.78-18.53 | 18 (16.67%) | 10.19-25.06 | 9 (11.25%) | 5.28-20.28 |
| **Multiple gestation** | 4  (4.49%) | 1.24-11.11 | 57 (13.16%) | 10.12-16.72 | 26 (10.66%) | 7.08-15.22 | 19 (11.05%) | 6.78-16.71 | 11 (12.50%) | 6.41-21.27 | 8  (7.41%) | 3.25-14.07 | 7  (8.75%) | 3.59-17.20 |
| **NRFHR** | 17 (19.10%) | 11.54-28.81 | 78 (18.01%) | 14.51-21.96 | 50 (20.49%) | 15.61-26.11 | 24 (13.95%) | 9.15-20.05 | 15 (17.05%) | 9.87-26.55 | 21 (19.44%) | 12.46-28.17 | 13 (16.25%) | 8.95-26.18 |
| **Malpresentation** | 28 (31.46%) | 22.03-42.17 | 112 (25.87%) | 21.80-30.26 | 36 (14.75%) | 10.55-19.84^□^ | 35 (20.35%) | 14.60-27.15 | 13 (14.77%) | 8.11-23.94 | 13 (12.04%) | 6.57-19.70^□^ | 7  (8.75%) | 3.59-17.20^□^ |
| **past uterine surgery** | 0  (0.00%) | 0.00-4.06 | 3  (0.69%) | 0.14-2.01 | 6  (2.46%) | 0.91-5.28 | 3  (1.74%) | 0.36-5.01 | 1  (1.14%) | 0.03-6.17 | 0  (0.00%) | 0.00-3.36 | 2  (2.50%) | 0.30-8.74 |
| **Cord prolapse** | 0  (0.00%) | 0.00-4.06 | 0  (0.00%) | 0.00-0.85 | 2  (0.82%) | 0.10-2.93 | 0  (0.00%) | 0.00-2.12 | 0  (0.00%) | 0.00-4.11 | 0  (0.00%) | 0.00-3.36 | 0  (0.00%) | 0.00-4.51 |
| **Chorioamnionitis** | 0  (0.00%) | 0.00-4.06 | 1  (0.23%) | 0.01-1.28 | 0  (0.00%) | 0.00-1.50 | 1  (0.58%) | 0.01-3.20 | 0  (0.00%) | 0.00-4.11 | 0  (0.00%) | 0.00-3.36 | 0  (0.00%) | 0.00-4.51 |
| **Failed operative vaginal delivery** | 1  (1.12%) | 0.03-6.10 | 9  (2.08%) | 0.95-3.91 | 2  (0.82%) | 0.10-2.93 | 4  (2.33%) | 0.64-5.85 | 1  (1.14%) | 0.03-6.17 | 1  (0.93%) | 0.02-5.05 | 1  (1.25%) | 0.03-6.77 |
| **Failed induction of labor** | 7  (7.87%) | 3.22-15.54 | 24 (5.54%) | 3.58-8.14 | 24 (9.84%) | 6.40-14.28 | 14 (8.14%) | 4.52-13.28 | 9 (10.23%) | 4.78-18.53 | 11 (10.19%) | 5.20-17.49 | 13 (16.25%) | 8.95-26.18^∆^ |
| **Suspected macrosomia** | 1  (1.12%) | 0.03-6.10 | 13 (3.00%) | 1.61-5.08 | 11 (4.51%) | 2.27-7.92 | 12 (6.98%) | 3.66-11.87 | 8  (9.09%) | 4.01-17.13 | 6  (5.56%) | 2.07-11.70 | 10 (12.50%) | 6.16-21.79^∆^ |
| **Fetal condition** | 2  (2.25%) | 0.27-7.88 | 7  (1.62%) | 0.65-3.30 | 2  (0.82%) | 0.10-2.93 | 1  (0.58%) | 0.01-3.20 | 1  (1.14%) | 0.03-6.17 | 2  (1.85%) | 0.23-6.53 | 0  (0.00%) | 0.00-4.51 |
| **Placenta previa or tumor previa** | 1  (1.12%) | 0.03-6.10 | 3  (0.69%) | 0.14-2.01 | 4  (1.64%) | 0.45-4.14 | 4  (2.33%) | 0.64-5.85 | 1  (1.14%) | 0.03-6.17 | 0  (0.00%) | 0.00-3.36 | 0  (0.00%) | 0.00-4.51 |
| **Placental abruption** | 0  (0.00%) | 0.00-4.06 | 3  (0.69%) | 0.14-2.01 | 2  (0.82%) | 0.10-2.93 | 1  (0.58%) | 0.01-3.20 | 0  (0.00%) | 0.00-4.11 | 1  (0.93%) | 0.02-5.05 | 0  (0.00%) | 0.00-4.51 |
| **Patient's request- no medical indication** | 0  (0.00%) | 0.00-4.06 | 4  (0.92%) | 0.25-2.35 | 2  (0.82%) | 0.10-2.93 | 3  (1.74%) | 0.36-5.01 | 2  (2.27%) | 0.28-7.97 | 2  (1.85%) | 0.23-6.53 | 3  (3.75%) | 0.78-10.57 |
| **Other indication** | 18 (20.22%) | 10.12-36.09 | 69 (15.94%) | 11.81-21.28 | 53 (21.72%) | 15.55-29.94 | 25 (14.53%) | 8.81-23.53 | 17 (19.32%) | 10.15-34.52 | 25 (23.15%) | 13.48-37.38 | 15 (18.75%) | 9.25-34.92 |

BMI- body mass index; N- number; CI- 95% confidence interval; NRFHR- non reassuring fetal heart rate.
∆- higher than Normal weight 1; ▲- higher than Normal weight 1 and Normal weight 2; □- lower than Normal weight 1; ■- lower than Normal weight 1 and Normal weight

**Supplementary Table 14: Distribution of the population as a whole and of the subgroup of healthy parturients into BMI groups based on pre-pregnancy BMI and BMI at the end of pregnancy.**

|  | | | **BMI groups** | | | | | | |  |
| --- | --- | --- | --- | --- | --- | --- | --- | --- | --- | --- |
|  |  |  | **Under-weight** | **Normal Weight 1** | **Normal Weight 2** | **Over-weight 1** | **Over-weight 2** | **Obesity** | **Morbid Obesity** | **Total  N (%^C^)** |
| **Study population** | **BMI at the beginning of index pregnancy (kg/m^2^)** | N | 1,074 | 5,473 | 2.957 | 1,734 | 990 | 1,039 | 459 | 13,726 (100%) |
|  |  | %^A^ | 100% | 100% | 100% | 100% | 100% | 100% | 100% |  |
|  |  | %^B^ | 7.82% | 39.87% | 21.54% | 12.63% | 7.21% | 7.57% | 3.34% |  |
|  | **BMI at the end of index pregnancy (kg/m^2^)** | N | 28 | 903 | 2,186 | 2,918 | 2,449 | 2,604 | 1,077 | 12,165 (88.63%) |
|  |  | %^A^ | 100% | 100% | 100% | 100% | 100% | 100% | 100% |  |
|  |  | %^B^ | 0.23% | 7.42% | 17.97% | 23.99% | 20.13% | 21.41% | 8.85% |  |
| **Healthy parturients** | **BMI at the beginning of index pregnancy (kg/m^2^)** | N | 942 | 4,599 | 2,424 | 1,369 | 803 | 772 | 334 | 11,243 (81.91%) |
|  |  | %^A^ | 87.71% | 84.03% | 81.97% | 78.95% | 81.11% | 74.30% | 72.77% |  |
|  |  | %^B^ | 8.38% | 40.91% | 21.56% | 12.18% | 7.14% | 6.87 | 2.97 |  |
|  | **BMI at the end of index pregnancy (kg/m^2^)** | N | 23 | 776 | 1,853 | 2,432 | 2,000 | 2,083 | 793 | 9,960 (72.56%) |
|  |  | %^A^ | 82.14% | 85.94% | 84.77% | 83.34% | 81.67% | 79.99% | 73.63% |  |
|  |  | %^B^ | 0.23% | 7.79% | 18.60% | 24.42% | 20.08% | 20.91% | 7.96% |  |

BMI- body mass index; N- number; %- percentage; kg- kilogram; m- meter.
^A^ percentage of the BMI group.
^B^ percentage of the index study population.
^C^ percentage of the original study population (i.e., fulfilling eligibility criteria).

**Supplementary Figure 15:** Correlation between pre-pregnancy BMI and increase in BMI during pregnancy in the study population.


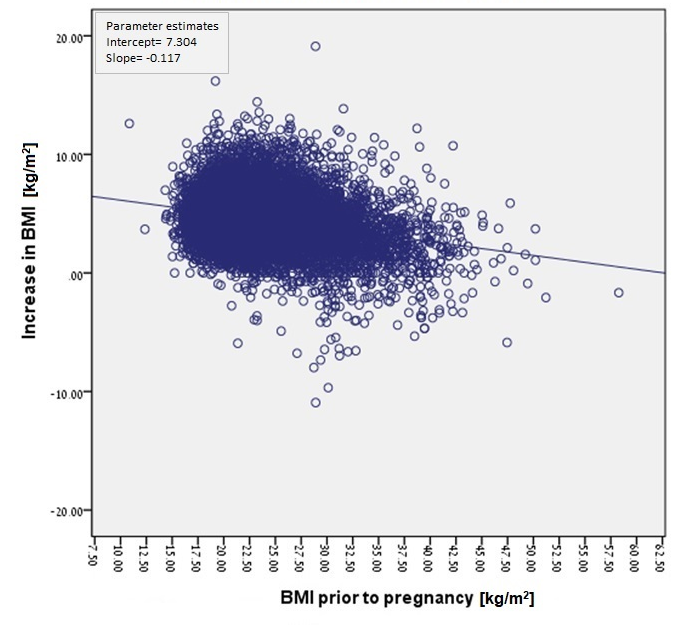


As pre-pregnancy weight and BMI increased, both relative and absolute maternal weight gain during pregnancy decreased, with an average weight gain of 12.9±5.2 kilograms in women who started their pregnancy with normal BMI and 6.1±7.1 kilograms in women who were morbidly obese at the beginning of their pregnancy (Supplementary Table 6). A similar correlation was observed between pre-pregnancy BMI and the absolute increase in BMI by the end of pregnancy (shown here).

**Supplementary Figure 16:** Correlation between pre-pregnancy BMI and increase in BMI during pregnancy in healthy parturients.


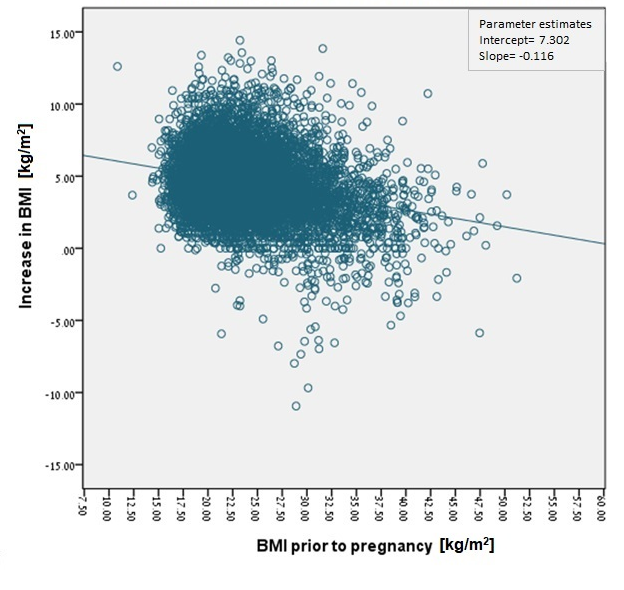


As pre-pregnancy weight and BMI increased, both relative and absolute maternal weight gain during pregnancy decreased, with an average weight gain of 13.0±5.1 kilograms in women who started their pregnancy with normal BMI and 5.9±7.0 kilograms in women who were morbidly obese at the beginning of their pregnancy (data not presented). A similar correlation was observed between pre-pregnancy BMI and the absolute increase in BMI by the end of pregnancy (shown here).
